# Elemental Constituents of Particulate Matter and Newborn’s Size in Eight European Cohorts

**DOI:** 10.1289/ehp.1409546

**Published:** 2015-06-05

**Authors:** Marie Pedersen, Ulrike Gehring, Rob Beelen, Meng Wang, Lise Giorgis-Allemand, Anne-Marie Nybo Andersen, Xavier Basagaña, Claire Bernard, Marta Cirach, Francesco Forastiere, Kees de Hoogh, Regina Gražulevičvienė, Olena Gruzieva, Gerard Hoek, Aleksandra Jedynska, Claudia Klümper, Ingeborg M. Kooter, Ursula Krämer, Jaakko Kukkonen, Daniela Porta, Dirkje S. Postma, Ole Raaschou-Nielsen, Lenie van Rossem, Jordi Sunyer, Mette Sørensen, Ming-Yi Tsai, Tanja G. M. Vrijkotte, Michael Wilhelm, Mark J. Nieuwenhuijsen, Göran Pershagen, Bert Brunekreef, Manolis Kogevinas, Rémy Slama

**Affiliations:** 1 Centre for Research in Environmental Epidemiology (CREAL), Barcelona, Spain; 2 CIBER Epidemiología y Salud Pública (CIBERESP), Madrid, Spain; 3 Universitat Pompeu Fabra, Barcelona, Spain; 4 Team of Environmental Epidemiology Applied to Reproduction and Respiratory Health, IAB (Institut Albert Bonniot), INSERM (National Institute of Health and Medical Research), U823, Grenoble, France; 5 Institute for Risk Assessment Sciences, Utrecht University, Utrecht, the Netherlands; 6 Department of Environmental and Occupational Health Sciences, University of Washington, Seattle, Washington, USA; 7 University Joseph Fourier, Grenoble, France; 8 Department of Social Medicine, University of Copenhagen, Copenhagen, Denmark; 9 Department of Epidemiology, Lazio Regional Health Service, Rome, Italy; 10 Department of Epidemiology and Public Health, Swiss Tropical & Public Health Institute, Basel, Switzerland; 11 University of Basel, Basel, Switzerland; 12 Department of Environmental Sciences, Vytauto Didziojo Universitetas, Kaunas, Lithuania; 13 Institute of Environmental Medicine, Karolinska Institutet, Stockholm, Sweden; 14 TNO (Netherlands Organisation for Applied Scientific Research), Utrecht, the Netherlands; 15 Institut für umweltmedizinische Forschung, Heinrich-Heine-Universität Düsseldorf, Düsseldorf, Germany; 16 Finnish Meteorological Institute, Helsinki, Finland; 17 Groningen Research Institute for Asthma and COPD, and; 18 Department of Pulmonary Diseases, University Medical Center Groningen, University of Groningen, Groningen, the Netherlands; 19 Danish Cancer Society Research Centre, Copenhagen, Denmark; 20 Julius Center for Health Sciences and Primary Care, University Medical Center Utrecht, Utrecht, the Netherlands; 21 IMIM (Hospital del Mar Research Institute), Barcelona, Spain; 22 Department of Public Health, Academic Medical Centre, University of Amsterdam, Amsterdam, the Netherlands; 23 Department of Hygiene, Social and Environmental Medicine, Ruhr-University Bochum, Bochum, Germany; 24 Department of Occupational and Environmental Health, Stockholm County Council, Stockholm, Sweden

## Abstract

**Background:**

The health effects of suspended particulate matter (PM) may depend on its chemical composition. Associations between maternal exposure to chemical constituents of PM and newborn’s size have been little examined.

**Objective:**

We aimed to investigate the associations of exposure to elemental constituents of PM with term low birth weight (LBW; weight < 2,500 g among births after 37 weeks of gestation), mean birth weight, and head circumference, relying on standardized fine-scale exposure assessment and with extensive control for potential confounders.

**Methods:**

We pooled data from eight European cohorts comprising 34,923 singleton births in 1994–2008. Annual average concentrations of elemental constituents of PM ≤ 2.5 and ≤ 10 μm (PM_2.5_ and PM_10_) at maternal home addresses during pregnancy were estimated using land-use regression models. Adjusted associations between each birth measurement and concentrations of eight elements (copper, iron, potassium, nickel, sulfur, silicon, vanadium, and zinc) were calculated using random-effects regression on pooled data.

**Results:**

A 200-ng/m^3^ increase in sulfur in PM_2.5_ was associated with an increased risk of LBW (adjusted odds ratio = 1.36; 95% confidence interval: 1.17, 1.58). Increased nickel and zinc in PM_2.5_ concentrations were also associated with an increased risk of LBW. Head circumference was reduced at higher exposure to all elements except potassium. All associations with sulfur were most robust to adjustment for PM_2.5_ mass concentration. All results were similar for PM_10_.

**Conclusion:**

Sulfur, reflecting secondary combustion particles in this study, may adversely affect LBW and head circumference, independently of particle mass.

**Citation:**

Pedersen M, Gehring U, Beelen R, Wang M, Giorgis-Allemand L, Andersen AM, Basagaña X, Bernard C, Cirach M, Forastiere F, de Hoogh K, Gražulevičienė R, Gruzieva O, Hoek G, Jedynska A, Klümper C, Kooter IM, Krämer U, Kukkonen J, Porta D, Postma DS, Raaschou-Nielsen O, van Rossem L, Sunyer J, Sørensen M, Tsai MY, Vrijkotte TG, Wilhelm M, Nieuwenhuijsen MJ, Pershagen G, Brunekreef B, Kogevinas M, Slama R. 2016. Elemental constituents of particulate matter and newborn’s size in eight European cohorts. Environ Health Perspect 124:141–150; http://dx.doi.org/10.1289/ehp.1409546

## Introduction

Low birth weight (LBW; birth weight < 2,500 g) is a predictor of infant morbidity and mortality. The evidence for associations between exposure to airborne particulate matter (PM) and LBW is growing (Fleischer et al. 2014; Pedersen et al. 2013a; Stieb et al. 2012). Recent meta-analyses show heterogeneity across studies conducted in different areas (Dadvand et al. 2013; Sapkota et al. 2012; Stieb et al. 2012). Many of these studies have also examined continuous birth weight, and some point toward an association between PM and mean birth weight adjusted for gestational age (Pedersen et al. 2013a; Stieb et al. 2012).

PM is a complex mixture of solid matter and liquid droplets made up of a number of components, including elemental carbon, metals, organic chemicals, acids, and soil material, which vary in composition by place and time (Bell et al. 2007). The health effects of PM may depend on its origin and chemical composition (Kelly and Fussell 2012; Stanek et al. 2011).

Associations of the composition of PM with an aerodynamic diameter of ≤ 2.5 μm (PM_2.5_) with LBW and/or birth weight were investigated in four large study populations from the United States (Basu et al. 2014; Bell et al. 2010, 2012; Darrow et al. 2011; Ebisu and Bell 2012) with mixed findings for birth weight in particular. For example, nickel (Ni) has been found to be associated with a reduction in birth weight in two of these studies (Basu et al. 2014; Bell et al. 2010), but not in a third study (Ebisu and Bell 2012). Because the elemental composition of PM differs by location, studies of PM constituents and LBW in other study areas are warranted. Moreover, these previous studies relied on exposure estimates based on data from the nearest regulatory air quality monitor, which do not capture within-city exposure contrasts adequately, possibly resulting in misclassification of exposure and reduced risk estimates (Woodruff et al. 2009). None of these studies examined associations between particulate constituents and birth head circumference, which has been associated with cognitive ability (Heinonen et al. 2008) and may, as we have shown, be influenced by PM_2.5_ and PM with an aerodynamic diameter ≤ 10 μm (PM_10_) mass concentration (Pedersen et al. 2013a).

Recently, land use regression (LUR) models have also been developed for eight *a priori* selected elements in both PM_2.5_ and PM_10_ in 20 study areas across Europe (de Hoogh et al. 2013) where birth cohorts had been conducted (Pedersen et al. 2013a), providing the opportunity to study the impact of PM composition on offspring measurements at birth in European populations. A high content of PM in each of these elements is to some extent characteristic of a different source. For example, brake linings tend to increase copper (Cu) and iron (Fe) concentrations; tire wear, zinc (Zn); residual oil combustion associated with areas with shipping, oil heating, and/or sizable industries is associated with Ni and vanadium (V) concentrations; biomass burning with potassium (K); secondary combustion pollution in long-range transport with sulfur (S), and crustal materials with silicon (Si) (Viana et al. 2008).

In the present study, we estimated the impact of exposures to these eight PM constituents on term LBW, birth weight, and birth head circumference in a large European study population with standardized fine-scale exposure assessment and extensive control for potential confounders.

## Methods

*Study population*. We pooled data from eight European mother–child cohorts conducted in areas where exposure to elemental composition of PM was assessed as part of the TRANSPHORM (Transport-related Air Pollution and Health impacts—Integrated Methodologies for Assessing Particulate Matter) project (de Hoogh et al. 2013): BAMSE–Sweden (Child, Allergy, Environment, Stockholm, Epidemiology; four centers close to Stockholm), DNBC–Denmark (Danish National Birth Cohort; Copenhagen), KANC–Lithuania (Kaunas neonatal cohort; Kaunas), ABCD (Amsterdam Born Children and their Development; Amsterdam), and PIAMA (Prevention and Incidence of Asthma and Mite Allergy; three centers)–the Netherlands, DUISBURG–Germany (Duisburg), GASPII–Italy (Gene and Environment: Prospective Study on Infancy in Italy; Rome), and INMA–Spain (INfancia y Medio Ambiente; Childhood and Environment; Sabadell). The cohorts, which were all part of the ESCAPE (European Study of Cohorts for Air Pollution Effects) project, have been previously described (Pedersen et al. 2013a). Four of the cohorts that were included in our previous study (Pedersen et al. 2013a)—namely MoBa (Norwegian Mother and Child Cohort Study)–Norway, Generation R–the Netherlands, APREG–Hungary (Air pollution and Pregnancy outcomes), and Rhea–Greece—were not included in the present analysis because exposure modeling and/or exposure assignment to the participants’ addresses was not performed in these study areas.

The study population included 34,923 singleton deliveries between 1994 and 2008. Detailed information on individual characteristics was obtained through interviews of the mother and self-administrated questionnaires during the pregnancy in most cohorts. Data from each cohort were harmonized and pooled centrally. Further information on the study design and the specific eligibility criteria applied in the baseline cohorts for the participation of mothers is summarized in Supplemental Material, Table S1. Ethical approval was obtained from the ethics committee in each country. Written informed consent was obtained from all participating women.

*Birth outcomes.* We defined term LBW among births occurring after 37 weeks of gestation as weight < 2,500 g. We also studied mean birth weight among term births and birth head circumference among all births as in our previous study (Pedersen et al. 2013a). Gestational age, birth weight, birth head circumference, sex, and mode of delivery were obtained from birth records or, for the PIAMA cohort, parental reports.

Gestational age (weeks) was defined as the interval between the start of the last menstrual period and delivery when possible (53% of births) (Slama et al. 2008). Ultrasound-based estimation (10%) was used only if last menstrual period was unavailable; when this was not possible, we used the estimates from the obstetrician (37%), which is usually ultrasound based.

*Air pollution exposure assessment.* We estimated exposure to PM elements in two size fractions (PM_2.5_ and PM_10_), using standardized methods developed within the ESCAPE and TRANSPHORM projects and relying on a LUR approach (de Hoogh et al. 2013).

Briefly, PM_2.5_ and PM_10_ concentrations in outdoor air were measured during three 2-week periods in summer, winter, and an intermediate season within 1 year at multiple sites (20–40) within each study area. Sites were selected to represent spatial variation of air pollution in the residential areas of the participants. Measurements were performed during 2008–2011 (see Supplemental Material, Table S1). PM_2.5_ and PM_10_ filters were weighed before and after each measurement centrally at IRAS (Institute for Risk Assessment Sciences), Utrecht University, and were then sent to Cooper Environmental Services (Portland, OR, USA) to detect elements. All filters were analyzed for elemental composition using X-ray Fluorescence (XRF) (de Hoogh et al. 2013). The three measurements were then averaged, adjusting for temporal trends using data from a continuous reference site (de Hoogh et al. 2013). The selected elements had a high percentage of well-detected samples, and a good precision of measurements (de Hoogh et al. 2013).

Annual mean concentrations of ambient elemental concentrations, PM_2.5_, and PM_10_ were estimated at the maternal home addresses during pregnancy with area-specific LUR models (de Hoogh et al. 2013; Eeftens et al. 2012). If no significant predictors could be included in a LUR model, we did not estimate cohort exposures, because the center-specific estimates would be the same for all cohort members from the area. For this reason, there were no Fe PM_25_, K PM_10_, Ni PM_2.5_, S PM_2.5_, S PM_10_, or Zn PM_2.5_ exposures estimated for the participants from the KANC cohort; K PM_2.5_ could not be estimated for the DUISBURG cohort, and Ni PM_2.5_ was missing for the BAMSE cohort. Because of lack of elemental data from routine monitoring stations in the study areas, we were unable to back-extrapolate the annual average elemental concentrations estimated by the LUR models to the time period of each individual pregnancy. Data from routine monitoring stations were used to temporally adjust the annual PM_2.5_ and PM_10_ LUR estimates to the periods corresponding to each individual pregnancy (Pedersen et al. 2013a). We accounted for changes of home address during pregnancy when the date of moving was known and mass concentration estimates was available at the new address.

*Statistical analysis.* We followed the same analysis plan as in our previous study (Pedersen et al. 2013a). Pooled analyses were conducted using mixed models including a random effect for center. Odds ratios (ORs) with 95% confidence intervals (CIs) for associations between exposure to air pollution and term LBW were estimated for fixed exposure increments using logistic regression models (i.e., xtlogit function in Stata S.E. version 12.1; StataCorp, College Station, TX, USA). Linear regression models were used for birth weight (grams) and birth head circumference (centimeters; xtmixed function).

Crude and adjusted models were examined. Adjustment variables were selected *a priori*; these were gestational age (weeks, continuous and quadratic terms), sex, parity (0, 1, ≥ 2), maternal height (centimeters), prepregnancy weight (broken stick model with a knot at 60 kg), mean number of cigarettes smoked during second trimester of gestation (cigarettes/day), maternal age (years), maternal education (cohort-specific definition of low, middle, high), and season of conception (January–March, April–June, July–September, October–December).

Each exposure variable was entered separately as a continuous variable in regression models.

Because of the correlations between some elemental constituents and particle mass, associations with elements may be biased and instead reflect relationships with particle mass. We therefore also performed analyses with two-pollutant models for each element adjusted for particle mass (PM_2.5_, PM_10_), other elements or traffic density on nearest street (vehicles per day), separately. We used variance inflation factors (VIF) diagnostics to assess collinearity problems, low values of VIF (< 5) being considered to be indicative that collinearity concerns are minor. We report two-pollutant models for pairs of pollutants selected from the results of the one-pollutant models.

Sensitivity analyses included *a*) restriction to women who did not change home address during pregnancy (assuming that residential mobility could result in exposure misclassification); *b*) restriction to areas where exposure models had the highest predictive value (defined as a cross-validation *R*^2^ for the LUR exposure model above 0.6); *c*) restriction to women from cohorts other than the DNBC, which accounted for half of the overall study population; *d*) restriction to women who participated with one pregnancy only (*n* = 34,088); *e*) further adjustment for maternal nationality (native vs. other) and self-reported maternal exposure to secondhand smoke (SHS) during pregnancy (no, yes); *f*) analyses stratified on maternal nationality (defined as above), maternal age (< 25, 25–35, > 35 years), sex, parity (0, ≥ 1), maternal active smoking (no, yes), maternal education (low, middle, high), or season of conception, to examine potential effect measure modification; and finally, we repeated analyses on term LBW and birth weight for the smaller study population with information on birth head circumference.

We tested for the effect of meteorological factors (ambient temperature, humidity, and atmospheric pressure, averaged over the full pregnancy and each trimester) on the birth outcomes of interest to decide whether adjustment was warranted; both linear and restricted cubic spline coding were considered. In sensitivity analyses, LBW and birth weight models were additionally adjusted for atmospheric pressure averaged over the full pregnancy, and head circumference was adjusted for average temperature (both fitted as a restricted cubic spline); we did not adjust for other meteorological factors because there were no evidence of associations (*p* > 0.05) between these factors and the outcomes assessed in the present study population.

We created exposure variables to estimate between-center and within-center exposure effects (Jerrett et al. 2008). To determine whether exposures could be assessed at the individual level across the entire range, we tested for significant differences between the estimated effects of center-aggregated means (referred to as between-center effects) and the estimated effects of the individually assigned exposures deviated from those center means (within-center effects).

Finally, we applied a false discovery rate (FDR) correction to correct for multiple testing (Benjamini and Yekutieli 2001) and chose an alpha level of 5% (two-sided) to define statistical significance.

## Results

Air pollution exposure was estimated for 34,923 mother–child pairs from eight cohorts in seven European countries (Table 1). Demographic and other characteristics are given in Table 1; see also Supplemental Material, Figure S1. Most of the participating women were born in the country of the cohort in which they participated (ranging from 100% in DNBC to 62.2% in ABCD). The prevalence of term LBW was 1.2%, mean birth weight was 3,531 g, and mean head circumference was 40.0 cm.

**Table 1:** Study population characteristics (total *N* = 34,923).

Variable	*n* (%)	Mean ± SD
Country, city, cohort
Sweden, Stockholm, BAMSE	3,860 (11.1)
Denmark, Copenhagen, DNBC	17,550 (50.3)
Lithuania, Kaunas, KANC	633 (1.8)
The Netherlands, Amsterdam, ABCD; multiple sites, PIAMA	11,430 (32.7)
Germany, Duisburg, DUISBURG	194 (0.6)
Italy, Rome, GASPII	684 (2.0)
Spain, Sabadell, INMA	572 (1.6)
Maternal nationality (*n *= 34,030)^*a*^
Born in country of cohort	30,491 (89.6)
Born elsewhere	3,539 (10.4)
Maternal age (years, *n *= 34,905)		30.4 ± 4.5
Maternal education (*n *= 33,719)
Low	6,609 (19.6)
Middle	15,336 (45.5)
High	11,774 (34.9)
Parity (*n *= 34,874)
No children	18,074 (51.8)
1 child	12,520 (35.9)
≥ 2 children	4,280 (12.3)
Maternal prepregnancy weight (kg; *n *= 33,822)		65.2 ± 11.5
Maternal height (cm; *n *= 34,334)		168.5 ± 6.6
Maternal smoking during 2nd trimester (*n *= 34,895)
No	29,689 (85.1)
Yes	5,206 (14.9)
No. of cigarettes per day among smokers (*n *= 5,206)		7.2 ± 5.3
Exposure to secondhand smoke (*n *= 30,900)
No	16,460 (53.3)
Yes	14,440 (46.7)
Moved residence during pregnancy (*n *= 34,868)
No	29,357 (84.2)
Yes	5,511 (15.8)
Season of conception
January–March	7,612 (21.8)
April–June	7,265 (20.8)
July–September	9,477 (27.1)
October–December	10,569 (30.3)
Gestational ambient temperature (°C; *n *= 34,277)		9.0 ± 2.6
Gestational ambient humidity (%)		78.0 ± 4.1
Gestational atmospheric pressure (mBar; *n *= 34,290)^*b*^		1,010 ± 6
Sex
Boy	17,752 (50.8)
Girl	17,171 (49.2)
Birth weight (g) among term births (> 37 weeks; *n *= 33,416)		3,531 ± 550
Term low birth weight (< 2,500 g in term births)	409 (1.2)
Birth head circumference (cm; *n *= 23,840)^*c*^		35.2 ± 1.7
Gestational age (weeks; *n *= 33,416)		40.0 ± 1.8
< 37 weeks	1,507 (4.3)
^***a***^Total in specific variables may be < 34,923 because of missing values. Of the women, 2.4% who participated had more than one pregnancy. ^***b***^Not available from KANC. ^***c***^Not available from all and from KANC, ABCD, and DUISBURG.		

**Figure 1:**
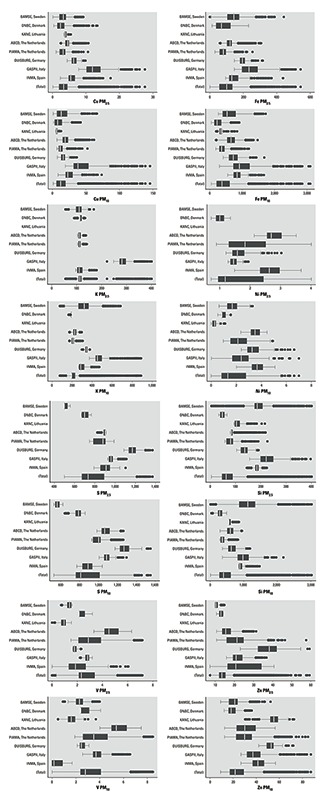
Distributions of exposure to PM constituents (ng/m^3^) by cohorts and for the pooled study population. Upper box plots for each element are for PM_2.5_ and the lower ones for PM_10_. The line in the middle of the box represents the median value, the ends of the box represent the 25th and 75th percentiles, and the whiskers indicate the variability outside the upper and lower quartiles (i.e., within 1.5 interquartile range of the lower quartile and upper quartile). Outliers are plotted as individual dots. Because no significant predictors could be included in the LUR models for a few study areas and pollutants, we were unable to estimate exposure to Fe PM_25_, K PM_10_, Ni PM_2.5_, S PM_2.5_, S PM_10_, and Zn PM_2.5_ for the participants from the KANC cohort; K PM_2.5_ was missing for the DUISBURG cohort; and Ni PM_2.5_ could not be estimated for the BAMSE cohort.

*Exposure levels.* Air pollution pregnancy exposure levels were on average 17.0 μg/m^3^ for PM_2.5_ and 26.9 μg/m^3^ for PM_10_ (Table 2; see also Supplemental Material, Figure S1). Distributions of estimated particle constituents varied between and within each cohort (Figure 1). The ratio of the between-center variance to within-center variance differed among the constituents, and for S in PM_2.5_ and PM_10_ the between/within area variance were 63 and 31, respectively (see Supplemental Material, Table S2). The highest concentrations and exposure gradients of Cu and Fe were observed for the Italian cohort (Figure 1). The Swedish and Danish cohorts were estimated to have the lowest mean exposure to S. As shown in Table 2, S, K, Fe, and Si contributed most to the mass of each particle mass fraction. Some elements were contained mainly in PM_2.5_ (S, Ni, V, Zn), others (Cu, Fe, Si) mainly in the coarse fraction (PM_10_–PM_2.5_). The eight selected elements represented 6% and 7% of the total mass of PM_2.5_ and PM_10_, respectively. Exposure to particle mass and particle constituents did not differ by maternal nationality (results not shown).

**Table 2:** Particle constituent concentrations and correlations between the elemental and total mass concentrations.

Exposure	PM_2.5_			PM_10_		
	*n*^*a*^	Mean ± SD	*r*^*b*^	*n*	Mean ± SD	*r*^*b*^
Mass concentration (μg/m^3^)	33,882	17.0 ± 4.7		33,882	26.9 ± 7.8	
Elemental concentration (ng/m^3^)
Copper (Cu)	34,923	3.4 ± 2.1	0.43	34,923	14.0 ± 10.6	0.54
Iron (Fe)	34,290	104.0 ± 57.4	0.17	34,923	435.3 ± 276.0	0.59
Potassium (K)	34,096	116.7 ± 27.4	0.20	34,290	224.3 ± 72.2	0.31
Nickel (Ni)	30,430	1.6 ± 0.8	0.67	34,923	1.8 ± 1.2	0.71
Sulfur (S)	34,290	753.9 ± 129.5	0.84	34,290	858.1 ± 171.7	0.84
Silicon (Si)	34,923	83.1 ± 54.4	0.02	34,923	489.7 ± 338.1	0.27
Vanadium (V)	34,923	2.8 ± 1.2	0.62	34,923	3.4 ± 1.5	0.66
Zinc (Zn)	34,290	14.8 ± 4.9	0.60	34,923	25.1 ± 10.9	0.61
^***a***^The numbers of participants are smaller for particle mass concentrations than for elemental concentrations because particle mass concentrations are pregnancy averages, and in some cases the routine monitoring data used to back-extrapolate particle mass concentrations were missing. Particle constituents are annual averages, and the numbers differ among particle constituents because we could not estimate all exposures in each study area. ^***b***^All Pearson correlations had a *p*-value < 0.001.						

Particle mass concentrations were correlated with some elemental concentrations as shown in Table 2, the correlation being highest between particle mass and S, lowest between particle mass and Si.

The correlation coefficients between PM constituents varied from –0.001 to 0.97 (see Supplemental Material, Table S3). The correlations between elemental and particle mass concentrations varied between cohorts, and the within-area correlations between mass and elements were smaller in each individual cohort than in the pooled sample (see Supplemental Material, Table S4).

*PM constituents and term low birth weight.* Significant ORs of term LBW were estimated for particle mass, S, and Ni in the PM_2.5_ fraction, and for particle mass and S in PM_10_ (Table 3). In two-pollutant models, the association of LBW with S in PM_2.5_ (OR = 1.24; *p* = 0.10) was stronger than the association with PM_2.5_ mass concentration (OR = 1.08; *p* = 0.42). This was also the case for S in PM_10_ (OR for S = 1.27; 95% CI: 1.03, 1.56; *p* = 0.02 vs. OR for PM_10_ = 1.00; 95% CI: 0.80, 1.25; *p* = 0.99) and Ni in PM_2.5_ (OR for Ni = 1.11; 95% CI: 0.94, 1.31; *p* = 0.21 vs. OR for PM_2.5_ = 1.05; 95% CI: 0.88, 1.24; *p* = 0.61). All VIFs were < 5 in the presented two-pollutant models.

**Table 3:** Adjusted associations between exposure to PM constituents and term LBW.

Model	PM_2.5_			PM_10_		
	*N*^*a*^	*n*^*b*^	OR (95% CI)^*c*^	*N*^*a*^	*n*^*b*^	OR (95% CI)^*c*^
Single-pollutant models
Mass	30,313	381	1.21 (1.08, 1.36)	30,313	381	1.22 (1.03, 1.45)
Cu	31,173	390	1.08 (0.81, 1.44)	31,173	390	1.13 (0.92, 1.39)
Fe	30,576	381	1.14 (0.92, 1.41)	31,173	390	1.06 (0.83, 1.36)
K	30,382	375	1.05 (0.82, 1.33)	30,576	381	0.90 (0.73, 1.11)
Ni	27,339	351	1.14 (1.00, 1.29)	31,173	390	1.29 (0.96, 1.75)
S	30,576	381	1.36 (1.17, 1.58)	30,576	381	1.27 (1.13, 1.43)
Si	31,173	390	0.83 (0.62, 1.12)	31,173	390	0.89 (0.71, 1.13)
V	31,173	390	1.12 (0.86, 1.44)	31,173	390	1.00 (0.72, 1.38)
Zn	30,576	381	1.23 (0.98, 1.54)	31,173	390	1.23 (0.98, 1.53)
Two-pollutant models
S adjusted for mass	29,716	372	1.24 (0.96, 1.61)	29,716	372	1.27 (1.03, 1.56)
Mass adjusted for S	29,716	372	1.08 (0.90, 1.30)	29,716	372	1.00 (0.80, 1.25)
Ni adjusted for mass	27,337	350	1.11 (0.94, 1.31)	30,313	381	1.14 (0.90, 1.43)
Mass adjusted for Ni	27,337	350	1.05 (0.88, 1.24)	30,313	381	1.15 (0.97, 1.37)
Zn adjusted for mass	29,716	372	1.11 (0.88, 1.40)	30,313	381	1.13 (0.90, 1.42)
Mass adjusted for Zn	29,716	372	1.19 (1.01, 1.40)	30,313	381	1.17 (0.97, 1.42)
S adjusted for Ni	27,339	351	1.08 (0.80, 1.46)	30,576	381	1.10 (0.83, 1.44)
Ni adjusted for S	27,339	351	1.10 (0.91, 1.33)	30,576	381	1.07 (0.85, 1.35)
S adjusted for Zn	30,576	381	1.39 (1.13, 1.70)	30,576	381	1.26 (1.10, 1.45)
Zn adjusted for S	30,576	381	0.96 (0.75, 1.23)	30,576	381	1.02 (0.82, 1.27)
^***a***^The number of subjects in each model. ^***b***^The number of term LBW cases in each model. ^***c***^Odds ratio (OR) and 95% confidence interval (CIs) for LBW (< 2,500 g) among term births (≥ 37 weeks of gestation) from pooled analyses using logistic regression models with random effect of center adjusted for gestational age, sex, parity, maternal height, prepregnancy weight, maternal active smoking during 2nd trimester, maternal age, maternal education, and season of conception per increments of 5 μg/m^3^ for PM_2.5_ mass; 5 ng/m^3^ for Cu PM_2.5_; 100 ng/m^3^ for Fe PM_2.5_; 50 ng/m^3^ for K PM_2.5_; 1 ng/m^3^ for Ni PM_2.5_; 200 ng/m^3^ for S PM_2.5_; 100 ng/m^3^ for Si PM_2.5_; 2 ng/m^3^ for V PM_2.5_; 10 ng/m^3^ for Zn PM_2.5_; 10 μg/m^3^ for PM_10_; 5 ng/m_3_ for Cu PM_2.5_; 10 μg/m^3^ for PM_10_; 20 ng/m^3^ for Cu PM_10_; 500 ng/m^3^ for Fe PM_10_; 100 ng/m^3^ for K PM_10_; 2 ng/m^3^ for Ni PM_10_; 200 ng/m^3^ for S PM_10_; 500 ng/m^3^ for Si PM_10_; 3 ng/m^3^ for V PM_10_; and 20 ng/m^3^ for Zn PM_10_.						

Sensitivity analyses supported an association between S and LBW both for the PM_2.5_ and PM_10_ fractions (see Supplemental Material, Table S5). The ORs changed very little after exclusion of the DNBC, of women who moved, and of women who participated with more than one pregnancy, and after additional adjustment for nationality and SHS. Excluding areas in which the LUR exposure models had the lowest *R*^2^ attenuated associations, but OR remained elevated and significant for PM_10_. Stratified analyses showed that the adjusted ORs for S (PM_2.5_ and PM_10_) were somewhat higher for women born elsewhere than in the country of the cohort, women who smoked, and women who gave birth for the first time, but none of these differences were significant. No significant differences (interaction *p* > 0.05) were seen by maternal education level, sex, maternal age, and season of conception. There was no consistent pattern of PM components having greater effects in boys or in girls (see Supplemental Material, Table S6).

The ORs remained elevated and significant for S in both PM_2.5_ and PM_10_ after further adjustment for atmospheric pressure (see Supplemental Material, Table S7).

The ORs for the association of LBW with the overall exposure to Cu, Ni, S, V, and Zn in both PM_2.5_ and PM_10_ (Table 3) were similar to the ORs for the association with the between-center exposure, but larger than ORs for the association with the within-center exposure (see Supplemental Material, Table S8). However, estimates for overall and within-center exposures were not significantly different according to the Wald test of equality of coefficients. For particle mass, especially for PM_2.5_, the within-center and between-center ORs were similar.

*PM constituents and mean birth weight.* In the analysis with birth weight as a continuous outcome, only PM_2.5_ mass and PM_2.5_ S were significantly associated with reduced birth weight; here, too, the association with PM_2.5_ S was stronger than with PM_2.5_ mass concentration (Table 4). Further adjustment for atmospheric pressure did not change the associations for S in PM_2.5_ considerably (mean change in birth weight: –40 g vs. –47 g in models further adjusted for atmospheric pressure; see Supplemental Material, Table S9). Opposite to the findings for LBW, the change in birth weight associated with the between-center exposures to particle mass, Ni, S, and Zn in both PM_2.5_ and PM_10_ were statistically significantly larger than the change associated with within-center exposures (see Supplemental Material, Table S10). There was no consistent pattern of the PM components having greater effects on birth weight or head circumference in boys than in girls (see Supplemental Material, Table S11).

**Table 4:** Adjusted associations between PM constituents and mean birth weight (g) in term births.

Model	PM_2.5_		PM_10_	
	*N*^*a*^	β**(95% CIs)^*b*^	*N*^*a*^	β**(95% CIs)^*b*^
Single-pollutant models
Mass	30,313	–16 (–29, –3)	30,313	–11 (–25, 2)
Cu	31,173	10 (–8, 27)	31,173	8 (–4, 19)
Fe	30,576	6 (–5, 16)	31,173	14 (1, 28)
K	30,382	11 (–11, 33)	30,576	14 (2, 27)
Ni	27,339	4 (–15, 22)	31,173	1 (–22, 24)
S	30,576	–40 (–64, –16)	30,576	–2 (–21, 17)
Si	31,173	26 (5, 48)	31,173	13 (–1, 27)
V	31,173	5 (–13, 23)	31,173	13 (–8, 35)
Zn	30,576	–4 (–21, 12)	31,173	8 (–6, 21)
Two-pollutant models
S adjusted for mass	29,716	–35 (–64, –7)	29,716	5 (–17, 27)
Mass adjusted for S	29,716	–6 (–22, 9)	29,716	–11 (–27, 5)
Ni adjusted for mass	27,337	7 (–13, 26)	30,313	–6 (–33, 20)
Mass adjusted for Ni	27,337	–8 (–23, 7)	30,313	7 (–26, 39)
Zn adjusted for mass	29,716	–1 (–18, 16)	30,313	14 (–1, 29)
Mass adjusted for Zn	29,716	–13 (–27, 1)	30,313	–18 (–34, –2)
Fe adjusted for mass	29,716	11 (–1, 22)	30,313	33 (16, 51)
Mass adjusted for Fe	29,716	–19 (–34, –5)	30,313	–34 (–52, –16)
Si adjusted for mass	30,313	36 (13, 60)	30,313	31 (14, 49)
Mass adjusted for Si	30,313	–20 (–34, –7)	30,313	–29 (–45, –13)
K adjusted for mass	29,522	12 (–11, 35)	29,716	24 (9, 39)
Mass adjusted for K	29,522	–14 (–28, 1)	29,716	–21 (–37, –6)
Fe adjusted for S	30,576	15 (4, 26)	30,576	19 (5, 33)
S adjusted for Fe	30,576	–56 (–80, –32)	30,576	–15 (–35, 6)
Si adjusted for S	30,576	33 (12, 54)	30,576	16 (2, 30)
S adjusted for Si	30,576	–45 (–69, –22)	30,576	–11 (–30, 8)
K adjusted for S	30,382	12 (–7, 30)	30,576	15 (2, 27)
S adjusted for K	30,382	–62 (–85, –37)	30,576	–5 (–24, 14)
S adjusted for Ni	27,339	–17 (–50, 16)	30,576	–6 (–33, 20)
Ni adjusted for S	27,339	7 (–50, 16)	30,576	7 (–26, 39)
S adjusted for Zn	30,576	–43 (–69, –16)	30,576	–1 (–21, 19)
Zn adjusted for S	30,576	4 (–14, 22)	30,576	–4 (–22, 13)
^***a***^The number of subjects in each model. ^***b***^Change in birth weight (in g) among term births (≥ 37 weeks of gestation) associated with exposure from pooled analyses using linear regression models with random effect of center. See Table 3 for adjustment factors. The following increments were used: 5 μg/m^3^ for PM_2.5_ mass; 5 ng/m^3^ for Cu PM_2.5_; 100 ng/m^3^ for Fe PM_2.5_; 50 ng/m^3^ for K PM_2.5_; 1 ng/m^3^ for Ni PM_2.5_; 200 ng/m^3^ for S PM_2.5_; 100 ng/m^3^ for Si PM_2.5_; 2 ng/m^3^ for V PM_2.5_; 10 ng/m^3^ for Zn PM_2.5_; 10 μg/m^3^ for PM_10_; 5 ng/m_3_ for Cu PM_2.5_; 10 μg/m^3^ for PM_10_; 20 ng/m^3^ for Cu PM_10_; 500 ng/m^3^ for Fe PM_10_; 100 ng/m^3^ for K PM_10_; 2 ng/m^3^ for Ni PM_10_; 200 ng/m^3^ for S PM_10_; 500 ng/m^3^ for Si PM_10_; 3 ng/m^3^ for V PM_10_; and 20 ng/m^3^ for Zn PM_10_.				

*PM constituents and birth head circumference.* The correlation coefficient between birth weight and head circumference is 0.60 in full-term infants (*n* = 23,024), which means that indeed birth weight does not explain all the variation in head circumference. All pollutants but K (PM_2.5_ and PM_10_) were associated with significant reductions in birth head circumference (Table 5).

**Table 5:** Adjusted associations between PM constituents and mean head circumference at birth (cm).

Model	PM_2.5_		PM_10_	
	*N*^*a*^	β^*b*^ (95% CIs)	*N*^*a*^	β^*b*^ (95% CIs)
Single-pollutant models
Mass	21,053	–0.25 (–0.30, –0.19)	21,053	–0.24 (–0.29, –0.18)
Cu	21,346	–0.31 (–0.37, –0.24)	21,346	–0.16 (–0.20, –0.12)
Fe	21,346	–0.19 (–0.23, –0.16)	21,346	–0.18 (–0.24, –0.13)
K	21,346	0.31 (0.23, 0.40)	21,346	0.04 (0.00, 0.09)
Ni	18,604	–0.60 (–0.71, –0.49)	21,346	–0.46 (–0.57, –0.36)
S	21,346	–0.79 (–0.92, –0.66)	21,346	–0.56 (–0.66, –0.47)
Si	21,346	–0.18 (–0.26, –0.09)	21,346	–0.12 (–0.17, –0.06)
V	21,346	–0.46 (–0.57, –0.36)	21,346	–0.49 (–0.60, –0.37)
Zn	21,346	–0.14 (–0.22, –0.05)	21,346	–0.27 (–0.34, –0.19)
Two-pollutant models
S adjusted for mass	21,053	–0.73 (–0.89, –0.57)	21,053	–0.51 (–0.63, –0.39)
Mass adjusted for S	21,053	–0.05 (–0.14, 0.02)	21,923	–0.05 (–0.13, 0.02)
Ni adjusted for mass	18,602	–0.49 (–0.61, –0.36)	21,053	–0.34 (–0.45, –0.22)
Mass adjusted for Ni	18,602	–0.14 (–0.22, –0.06)	21,053	–0.16 (–0.22, –0.10)
Zn adjusted for mass	21,053	0.03 (–0.06, 0.12)	21,053	–0.09 (–0.19, 0.01)
Mass adjusted for Zn	21,053	–0.26 (–0.32, –0.19)	21,053	–0.20 (–0.27, –0.13)
S adjusted for Ni	18,604	–0.64 (–0.80, –0.47)	21,346	–0.53 (–0.65, –0.41)
Ni adjusted for S	18,604	–0.31 (–0.44, –0.19)	21,346	–0.05 (–0.20, 0.09)
S adjusted for Zn	21,346	–0.92 (–1.06, –0.77)	21,346	–0.55 (–0.65, –0.44)
Zn adjusted for S	21,346	0.18 (0.08, 0.28)	21,346	–0.03 (–0.12, 0.06)
^***a***^The number of subjects in each model. Birth head circumference is not available from KANC, ABCD, and DUISBURG. ^***b***^Change in birth head circumference (cm) associated with exposure from pooled analyses using linear regression models with random effect of center adjusted for gestational age, sex, parity, maternal height, prepregnancy weight, maternal active smoking during 2nd trimester, maternal age, maternal education, season of conception, and pregnancy mean temperature. The following increments were used: 5 μg/m^3^ for PM_2.5_ mass; 5 ng/m^3^ for Cu PM_2.5_; 100 ng/m^3^ for Fe PM_2.5_; 50 ng/m^3^ for K PM_2.5_; 1 ng/m^3^ for Ni PM_2.5_; 200 ng/m^3^ for S PM_2.5_; 100 ng/m^3^ for Si PM_2.5_; 2 ng/m^3^ for V PM_2.5_; 10 ng/m^3^ for Zn PM_2.5_; 10 μg/m^3^ for PM_10_; 5 ng/m_3_ for Cu PM_2.5_; 10 μg/m^3^ for PM_10_; 20 ng/m^3^ for Cu PM_10_; 500 ng/m^3^ for Fe PM_10_; 100 ng/m^3^ for K PM_10_; 2 ng/m^3^ for Ni PM_10_; 200 ng/m^3^ for S PM_10_; 500 ng/m^3^ for Si PM_10_; 3 ng/m^3^ for V PM_10_; and 20 ng/m^3^ for Zn PM_10_.				

In two-pollutant models for head circumference, the associations with S (in both PM_2.5_ and PM_10_) and Ni (PM_2.5_ only) were stronger than the associations with PM mass concentration. Also the association for Ni PM_2.5_ remained statistically significant in two-pollutant models after adjustment for S PM_2.5_. The association with Ni in PM_10_ was null after adjustment for S in PM_10_, whereas associations with S in PM_10_ and PM_2.5_ persisted after adjustment for Ni.

For all outcomes, the effects estimates of both S and Zn in PM_2.5_ do not change much after adjustment for traffic density (results not shown). Further adjustment for temperature and atmospheric pressure averaged during the full pregnancy did not change any of the associations (see Supplemental Material, Table S12). The effects of between- and within-center exposures on head circumference were significantly different for some but not all pollutants (see Supplemental Material, Table S13). For this outcome, the overall effects reflected more the effects of the within-center variations in exposures. The within-center effects on head circumference were significantly larger than the between-center effects for particle mass, Ni, S, and V in PM_2.5_ as well as S and V in PM_10_. For Zn, the between-center effects were stronger than the within-center effects. For K in PM_2.5_, there was a stronger positive association within-center.

The study population for head circumference analyses was smaller than the population for birth weight analyses (Table 1); in terms of S in PM_2.5_, the OR for term LBW in the full study population was 1.36 (95% CI: 1.17, 1.58), which was slightly smaller than the OR of 1.43 (95% CI: 1.14, 1.79) in the restricted study population with head circumference information. There was no difference in the associations of S in PM_2.5_ with birth weight (full population: –41 g; 95% CI: –69, –12 vs. restricted population: –40 g; 95% CI: –64, –16).

After we applied an FDR correction, the effects of Ni and Zn in PM_2.5_ lost their statistical significance for term LBW (FDR-corrected *p*-values, 0.35 and 0.55, respectively), but the effects of mass (PM_2.5_) and S (both PM fractions) remained significant. For birth weight, only the effect estimates of S in PM_2.5_ remained statistically significant, whereas for head circumference, all effects were significant, except for K in PM_10_ (results not shown).

## Discussion

We examined associations of eight elemental constituents in PM_2.5_ and PM_10_ with newborn’s size in eight cohorts from seven European countries. S in both PM_2.5_ and PM_10_ and Ni in PM_2.5_ were associated with an increased risk of term LBW, reduced birth weight, and smaller birth head circumference. The association between S and LBW was robust to adjustment for co-pollutants including particle mass as well as FDR correction. The particle mass effect estimates were reduced and became statistically nonsignificant after adjustment for S.

S in PM_2.5_ has been associated with increased ORs for LBW in two studies from the United States (Basu et al. 2014; Bell et al. 2010). The estimated 36% increase in LBW at term associated with a 200 ng/m^3^ for S in PM_2.5_ in our study is, however, larger than those reported by the previous studies; the recalculated ORs for a 200-ng/m^3^ increase in pregnancy mean exposure to S in PM_2.5_ were 1.05 (95% CI: 0.97, 1.13) (Bell et al. 2010) and 1.02 (95% CI: 1.00, 1.04) (Basu et al. 2014). Previous findings also support a reduction in mean birth weight associated with S in PM_2.5_; Basu et al. (2014) reported a statistical significant reduction, and Bell et al. (2010) reported a nonsignificant reduction in birth weight (the recalculated reduction for a 200-ng/m^3^ increase in pregnancy mean exposure to S in PM_2.5_ were –8 g (95% CI: –10, –7) and –2 g (95% CI: –7, 3), respectively, compared to –40 g (95% CI: –64, –16) in our study). The differences between ours and previous studies may be explained by differences in study design and the fact that the previous U.S. studies on elemental composition of PM relied on exposure estimates based on data from the nearest regulatory air quality monitor, which do capture temporal trends (which our model did not capture), but less efficiently capture within-city exposure contrasts, and may have resulted in misclassification of exposure. Moreover, we were able to estimate both the effects of between-center and within-center variations in air pollution concentrations. The nonsignificant differences of the effects of between center and within center exposure on LBW may be seen as indicating that both regional and local pollution contribute to the associations with LBW. Previous studies were not always able to consider known and suspected risk factors of LBW such as maternal stature, smoking, parity, atmospheric pressure, humidity, and season of conception. This is not a purely theoretical concern; as an illustration, in our study most elemental components were statistically significantly associated with birth weight in crude models and in models without adjustment for parity, but not in the most comprehensive models we used, which included parity.

Our comparison of between-center and within-center effects indicates that between-center differences in exposure contribute more to the reductions in mean birth weight than within-center exposure contrasts. These findings could reflect the smaller exposure contrasts within each center compared with the larger exposure contrasts derived from the pooled population (Figure 1) and perhaps also residual confounding by unmeasured factors.

Our findings on head circumference indicate that the within-center variation of these elements is important and that the reductions in head circumference were driven by within-center exposure contrasts for most pollutants except for K and Zn (see Supplemental Material, Table S13).

Head circumference has been associated with cognitive ability (Heinonen et al. 2008) and child intellectual quotient (Eriksen et al. 2013). No previous study has examined the associations between PM_2.5_ components and birth head circumference. In our study all the elemental components except K were significantly associated with smaller head circumference.

Previous birth outcome studies of PM components (Basu et al. 2014; Bell et al. 2010; Ebisu and Bell 2012) did not report results of two-pollutant models adjusted for particle mass. Disentangling the effects of some elements from the effects of particle mass and disentangling the effects of various elemental components is challenging because of the correlations among predicted concentrations, due to partly similar patterns and sources. However, we found no clear indication of co-linearity in the presented two-pollutant models. Results for S adjusted for particle mass concentration did not differ from those from the single-pollutant models. We also investigated whether the previously observed associations with PM_2.5_ (Pedersen et al. 2013a) were robust to adjustment for S, Ni, and Zn. The findings of the present study suggest that the effect estimates associated with particle mass for LBW, birth weight, and head circumference were reduced and became less significant in two-pollutant models, both for PM_2.5_ and PM_10_, after adjustment for either S or Ni, whereas the associations for particle mass were robust for adjustment for other elements (Tables 3–5). Zn, described as a marker for tire wear (Viana et al. 2008), was associated with LBW, suggesting that that non-exhaust component of traffic-related air pollution was associated with LBW, whereas Cu and Fe, other components of non-exhaust traffic elements, were not associated with LBW.

In our study, most of the variability in S exposure occurred between (rather than within) the study areas, and the spatial variation of S within areas was explained mostly by various traffic, land use, and residential density variables (de Hoogh et al. 2013). In Europe, a minor fraction of S in particulate form is part of vehicle exhausts, but particulate sulfate is determined mostly by secondary aerosol formation (Viana et al. 2008). Sulfate is mostly formed in the atmosphere by oxidation of gaseous sulfur dioxide (SO_2_) emissions [U.S. Environmental Protection Agency (EPA) 2004]. In our study, the between-area variation of sulfur concentrations is more likely to depend on sources such as energy production and distribution rather than traffic, given the small contribution of traffic to the overall sulfur emissions (European Environmental Agency 2014). Sulfate in PM can be transported over regional- or continental-scale distances, resulting in a regional background with typically small spatial variation in metropolitan areas (U.S. EPA 2004). However, the exposure to S in both PM_2.5_ and PM_10_ varied less within centers than between centers (Figure 1), and the ratio between-center variance divided by the within-center variance of exposure was higher than the ratio values of all other components and particle mass (see Supplemental Material, Table S2).

Maternal exposure to ambient PM and combustion-related air pollutant gases such as nitrogen dioxide (NO_2_) and SO_2_ has been associated with risk of LBW (Pedersen et al. 2013a; Stieb et al. 2012), although it is unclear whether the association with NO_2_ is explained partly by the association with PM (which is correlated with NO_2_) and LBW. Mechanisms for the association between air pollution exposure and pregnancy outcomes are not well understood, but several hypotheses exist (Kannan et al. 2006). Inhalation of particles can trigger maternal oxidative stress, lipid peroxidation, inflammation, changes in the blood system; damage vascular endothelium; and thereby decrease placental blood flow, disrupt transplacental oxygenation, cause placental oxidative stress and inflammation, and lead to intrauterine growth restriction (Kannan et al. 2006). Metals such as lead and cadmium have been found to be embryotoxic and teratogenic in a variety of animal species and may also influence human intrauterine growth (Bellinger 2005; Kippler et al. 2012). Genotoxic and epigenetic effects of air pollutants are also possible and may entail effects on fetal growth (Janssen et al. 2013; Pedersen et al. 2013b). Data on humans are limited, and more mechanistic studies on PM components are needed.

In addition to the ability to specify two-pollutant models and to estimate the between-center and within-center effects, the main strengths of our study are the standardized, comprehensive exposure assessment of multiple elements with a high percentage of detected samples (> 75%), and good precision of measurements in all eight cohorts; the harmonized and detailed information on potential confounders; and the large population spread over a large geographical area. Detailed information on individual characteristics (e.g., maternal stature, parity, nationality, education, active and passive smoking during pregnancy) was collected prospectively in a manner that enabled us to reduce potential biases through adjustment.

Studies on temporal stability for elemental PM concentrations in our study areas are yet to be performed. Spatial contrasts of NO_2_ have been shown to be stable over time in a study, which covered the Italian study area included in the present study (Cesaroni et al. 2012); but because we used annual LUR estimates to assess pregnancy exposures occurring 2–15 years earlier (with most birth cohorts starting in the early 2000s), we recognize the potential for exposure misclassification. The finding of NO_2_ stability over time may be applicable to traffic-related constituents such as Cu, Fe, and Zn, whereas there is no quantitative evidence for the other constituents, which derive in part from other sources. No evidence of significant differences among seasons has been found for PM_10_ Cu (de Hoogh et al. 2013), but seasonal variation has not been tested for the other components in our study areas. A higher mean concentration of S in summer compared with the winter mean concentration has been reported in the United States (Bell et al. 2007), whereas no clear seasonal variation was observed for S in either PM_2.5_ or PM_10_ in Barcelona, Spain (Minguillón et al. 2014). The performance of the LUR models varied between the study areas and was poor in certain areas for some constituents (de Hoogh et al. 2013). Excluding areas with lowest *R*^2^ of LUR attenuated associations, but ORs remained elevated and significant for PM_10_ (see Supplemental Material, Table S5). The adjusted OR for LBW associated with PM_2.5_ was 1.21 (95% CI: 1.08, 1.36) in the present study, which is similar to the OR of 1.18 (95% CI: 1.06, 1.33) from our previous study (Pedersen et al. 2013a). The corresponding reductions in birth weight and head circumference were slightly larger in the current study as compared with our previous study. These differences are attributable to the different study population, that is, the current study is based on 30,313 of the 50,151 (60.4%) mother–child pairs included in our previous study.

Our exposure assessment was limited to home address(es), and exposures elsewhere such as at work or during commuting were not estimated, because detailed information on time–activity patterns or personal measures were not available. We had information on work status during pregnancy for five out of the eight cohorts, but we did not perform sensitivity analyses restricted to nonworking women (*n* = 7,608) because nonworking women differed from the working group of women (*n* = 18,159) in terms of nationality, age, parity, smoking habits, and other risk factors (results not shown), and it was unknown whether these nonworking women spend more time at home than the working women. Incomplete information on residential mobility may introduce exposure misclassification. Most women (84%), however, did not move residence during pregnancy, and analyses restricted to women who did not change home address during pregnancy gave very similar results to those reported for the full study population.

We used a simple measure to identify infants with suboptimal growth as we restricted the analyses of LBW and change in birth weight to full-term infants (born after 37 completed weeks) in order to separate effects on fetal growth from effects on inadequate time to growth (preterm delivery). In our study we chose not to study small-for-gestational-age because different country-specific reference growth curves were applied in the different centers. Moreover, it is not straightforward to apply a common growth curve for quantification of the deviation in birth weight relative to the expected weight at birth to a study population enrolled from a large heterogeneous study area because fetal growth not only differs by gestational age but also by, for example, nationality, sex, parity, and maternal stature. For this reason we choose to focus on simple measures of newborn’s size at birth.

Finally, we acknowledge that because low birth weight is associated with altered growth patterns in childhood, it would be relevant to consider effects of air pollution on child weight and height at other time points in and after pregnancy, as done in a few studies (Fleisch et al. 2015; Jerrett et al. 2014; Ritz et al. 2014).

We investigated eight *a priori* selected elements in both the PM_2.5_ and PM_10_ fraction, so there might be issues related to multiple comparisons, and the correlation between different elements and the extent to which they can act as surrogate for the exposures causing the effect. Associations with S remained after applying an FDR, though other associations weakened. One should keep in mind that correction for multiple testing in situations like the present one, with several (related) end points and correlations between exposures, is complex (Bender and Lange 2001) and that the FDR approach that we have used assumes independence between covariates. We therefore rather interpreted results on the basis of consistency of effect estimates across elements and PM size fractions.

We conclude that S, reflecting secondary combustion particles in this study, may adversely affect LBW and birth head circumference, independent of particle mass.

## Supplemental Material

(1.3 MB) PDF
