# Supplementary material for: Elemental Constituents of Particulate Matter and Newborn’s Size in Eight European Cohorts
Source: Environ Health Perspect. 2015 Jun 5;124(1):141–50. doi: 10.1289/ehp.1409546 (PMC4710606; doi:10.1289/ehp.1409546)
Supplement: (1.3 MB) PDF [file ehp.1409546.s001.acco.pdf]

*Environ Health Perspect*
DOI: 10.1289/ehp.1409546

**Note to Readers:** *EHP* strives to ensure that all journal content is accessible to all readers. However, some figures and Supplemental Material published in *EHP* articles may not conform to 508 standards due to the complexity of the information being presented. If you need assistance accessing journal content, please contact ehp508@niehs.nih.gov. Our staff will work with you to assess and meet your accessibility needs within 3 working days.

# Supplemental Material

# Elemental Constituents of Particulate Matter and Newborn's Size in Eight European Cohorts

Marie Pedersen, Ulrike Gehring, Rob Beelen, Meng Wang, Lise Giorgis-Allemand, Anne-Marie Nybo Andersen, Xavier Basagaña, Claire Bernard, Marta Cirach, Francesco Forastiere, Kees de Hoogh, Regina Gražulevičienė, Olena Gruzieva, Gerard Hoek, Aleksandra Jedynska, Claudia Klümper, Ingeborg M. Kooter, Ursula Krämer, Jaakko Kukkonen, Daniela Porta, Dirkje S. Postma, Ole Raaschou-Nielsen, Lenie van Rossem, Jordi Sunyer, Mette Sørensen, Ming-Yi Tsai, Tanja G. M. Vrijkotte, Michael Wilhelm, Mark J. Nieuwenhuijsen, Göran Pershagen, Bert Brunekreef, Manolis Kogevinas, and Rémy Slama

### Table of Contents

**Table S1.** Mother-child cohort data collection.

**Table S1.** Mother-child cohort data collection – cont.

**Table S2.** Variance of between- vs. within-center variations for the elements.

**Table S3.** Correlations between PM constituents (ng/m$^3$).

**Table S4.** Correlations between elemental (ng/m$^3$) and total mass concentration of PM (μg/m$^3$) by cohort.

**Table S5.** Sensitivity and stratified analyses of sulfur on term LBW.

**Table S6.** Sex-stratified associations between exposure to PM constituents and term LBW.

**Table S7.** Associations between exposure to PM constituents and term LBW results from the study population with information on atmospheric pressure and pressure adjusted models.

**Table S8.** Associations between exposure to PM constituents and term LBW results from between centers and within centers models.

**Table S9.** Associations between exposure to PM constituents and birth weight results from the study population with information on atmospheric pressure and pressure adjusted models.

**Table S10.** Associations between exposure to PM constituents and birth weight results from between centers and within centers models.

**Table S11.** Sex-stratified associations between exposure to PM constituents, birth weight and head circumference.

**Table S12.** Associations between exposure to PM constituents and birth head circumference results from the study population with information on atmospheric pressure and temperature and pressure adjusted models.

**Table S13.** PM constituents and birth head circumference results from between centers and within centers models.

**Figure S1.** Distributions of $PM_{2.5}$, $PM_{10}$, maternal height, pre-pregnancy weight, birth weight, birth head circumference and gestational age by cohorts and for the pooled study population. The line in the middle of the box represents the median values, the ends of the box refer to the 25th and 75th percentiles and the ends of the whiskers indicate the variability outside the upper and lower quartiles (i.e., within 1.5 interquartile range of the lower quartile and upper quartile). Outliers are plotted as individual dots.

**Table S1.** Mother-child cohort data collection.

| Cohort | Country | Centre(s) | Inclusion and exclusion criteria N (n) | Methods of maternal information collection | Gestational age estimation | Pregnancy (Years) | Air sampling (Pollutants) (Years) |
|---|---|---|---|---|---|---|---|
| BAMSE<br><br>'Barn, Allergi, Miljo, Stockholm, Epidemiologi'<br>'Child, Allergy, Environment, Stockholm, Epidemiology' | Sweden | Jarfalla<br>Solna<br>Sundbyberg<br>Stockholm | All parents of newborn children during 1994-1996 living in the study area, with no plans of moving within 1 year after birth, sufficient Swedish language skills and no serious family diseases. Included only women with one child. N=4,089. Pregnancy exposure to air pollution is available (n=3,868). | Self-administrated questionnaires starting at 3 months after birth of the child. | US based estimation (89%) | 1994-1996 | PM + $NO_X$<br>2008-2009 |
| DNBC<br><br>'Bedre Sundhed for Mor og Barn Undersøgelsen'<br>'Danish National Birth Cohort' | Denmark | National | All pregnant women who attended general practitioner in week 6-12 of pregnancy, who wanted to carry their pregnancy to term, and had sufficient Danish language skills to complete interviews during 1996-2002. Same women may participate multiple times. N=101,042. Pregnancy exposure to air pollution is available for the greater Copenhagen (n=17,577). | Computerized telephone interviews starting in week 12 of pregnancy. | LMP and US based estimation available. | 1996-2002 | PM + $NO_X$<br>2009-2010 |
| KANC | Lithuania | Kaunas | All pregnant women who attended general practitioner in first trimester of pregnancy during 2007-2008.<br>Women who lived outside Kaunas municipality, had medical records of pregnancy induced hypertension and/or diabetes were excluded. Pregnancy exposure to air pollution is available (n=4,101). | Face-to-face interviews by trained nurse and telephone interviews during pregnancy. | Maternity unit based estimation in which LMP is combined with US. | 2007-2008 | PM + $NO_X$<br>2010-2011 |
| ABCD<br><br>'Amsterdam Born Children and Their Development' | The Netherlands | Amsterdam | All pregnant women who lived in Amsterdam and attended prenatal care in early pregnancy during 2003-2004. Excluded multiple, stillbirth. N=7,863. Pregnancy exposure to air pollution is available (n=7,592). | Self-administrated questionnaires starting in early pregnancy. | Maternity unit based estimation relying on US or LMP if US was unavailable (<10%). | 2003-2004 | PM + $NO_X$<br>2009-2010 |
| PIAMA<br><br>'Prevention and Incidence of Asthma and Mite Allergy' | The Netherlands | North<br>West<br>Middle | Pregnant women who attended prenatal healthcare in approximately 50 clinics located in different parts of the Netherlands. Atopic (n=1327) and non-atopic women (n=2,819) were included. Pregnancy exposure to air pollution is available (n=3,839). | Self-administrated questionnaires starting at the women's first visit to the prenatal health clinics, which usually takes place during the first trimester. | Maternity unit based estimation in which LMP is combined with US. | 1996-1997 | PM + $NO_X$<br>2009-2010 |

**Table S1.** Mother-child cohort data collection – cont.

| Cohort | Country | Centre(s) | Inclusion and exclusion criteria N (n) | Methods of maternal information collection | Gestational age estimation | Pregnancy (Years) | Air sampling (Pollutants) (Years) |
|---|---|---|---|---|---|---|---|
| DUIBURG | Germany | Duisburg | Pregnant women who lived in a predefined area of Duisburg. Healthy, German or Turkish speaking women without serious pregnancy and/or birth complications with birth at term (i.e. ≥38-42 weeks of gestation) to children without APGAR scores ≥8 and without congenital anomalies. N=232. Pregnancy exposure to air pollution is available (n=194). | Face-to-face interviews starting in early pregnancy. | Maternity unit based estimation in which LMP is combined with US. | 2000-2002 | PM + $NO_X$ 2008-2009 |
| GASPII | Italy | Rome | Women delivering at two hospitals in the area of the local health unit RME in the north of the city, resident in the same area, Italian speaking, aged more than 17 years. Out of the all women contacted, 55% responded, out the eligible women contacted 34% responded. Pregnancy exposure to air pollution is available (n=684). | Face-to-face the day after delivery. | LMP based estimation and maternity unit. | 2003-2004 | PM + $NO_X$ 2010-2011 |
| INMA<br>'INfancia y Medio Ambiente'<br>'Childhood and Environment' | Spain | Sabadell | Pregnant women who attended prenatal care 10-13 week of pregnancy. Included women had delivery and residence in the study areas, at least 16 years old, singleton pregnancies, no assisted reproduction and no communication problems.<br>Enrolment took place in Sabadell (n=657, 2004-2006). | Face-to-face interviews and self-administrated questionnaires starting early pregnancy. | LMP, US and maternity unit based estimation in which LMP is combined with US. US was used when the difference with the LMP and US estimation was ≥ 7 days (12% of the cases). | 2004-2006 | PM + $NO_X$ 2009-2010 |

N refers to total number of participation women at birth; n refers to the number of participation women living in the ESCAPE LUR exposure areas with sufficient data to estimate the exposures during pregnancy; $NO_X$ refers to nitrogen oxides; LMP refers to last menstrual period; PM refers to particulate matter and US refers to ultrasound.

**Table S2.** Variance of between- vs. within-center variations for the elements.

| | Between-center variance | Within-center variance | Between/within-center variances ratio |
|---|---|---|---|
| $PM_{2.5}$ | 37.1 | 2.8 | 13.2 |
| Cu | 7.7 | 1.7 | 4.4 |
| Fe | 2888.5 | 2142.2 | 1.3 |
| K | 2819.7 | 93.4 | 30.2 |
| Ni | 0.5 | 0.06 | 8.3 |
| S | 4507.8 | 713.4 | 63.4 |
| Si | 3268.8 | 472.8 | 6.9 |
| V | 1.4 | 0.3 | 5.7 |
| Z | 63.2 | 7.4 | 8.6 |
| $PM_{10}$ | 76.9 | 11.2 | 6.9 |
| Cu | 105.5 | 67.5 | 1.6 |
| Fe | 59156.9 | 30967.9 | 1.9 |
| K | 8215.9 | 1392.8 | 5.9 |
| Ni | 1.1 | 0.2 | 7.5 |
| S | 59338.7 | 1888.6 | 31.4 |
| Si | 114440.1 | 28941.9 | 4.0 |
| V | 2.0 | 0.4 | 4.9 |
| Z | 146.7 | 49.6 | 3.0 |

**Table S3.** Correlations between PM constituents (ng/m$^3$).

| $PM_{2.5}$ | Cu | Fe | K | Ni | S | Si | V | Cu | Fe | K | Ni | S | Si | V | Zn |
|---|---|---|---|---|---|---|---|---|---|---|---|---|---|---|---|
| Cu | | | | | | | | 0.82 | 0.78 | 0.60 | 0.53 | 0.48 | 0.46 | 0.40 | 0.59 |
| Fe | 0.77 | | | | | | | 0.76 | 0.76 | 0.55 | 0.42 | 0.19 | 0.56 | 0.28 | 0.52 |
| K | 0.61 | 0.33 | | | | | | 0.43 | 0.33 | 0.63 | 0.04 | 0.21 | 0.23 | 0.04 | 0.30 |
| Ni | 0.48 | 0.39 | -0.06 | | | | | 0.49 | 0.67 | 0.28 | 0.97 | 0.88 | 0.68 | 0.84 | 0.57 |
| S | 0.50 | 0.20 | 0.26 | 0.82 | | | | 0.38 | 0.36 | 0.02 | 0.69 | 0.95 | -0.10 | 0.65 | 0.61 |
| Si | 0.53 | 0.58 | 0.34 | 0.55 | -0.07 | | | 0.47 | 0.68 | 0.86 | 0.36 | -0.06 | 0.92 | 0.04 | 0.37 |
| V | 0.35 | 0.18 | -0.001 | 0.91 | 0.74 | -0.08 | | 0.39 | 0.38 | -0.07 | 0.80 | 0.84 | 0.006 | 0.95 | 0.32 |
| Zn | 0.44 | 0.24 | 0.33 | 0.36 | 0.66 | 0.17 | 0.27 | 0.34 | 0.39 | 0.25 | 0.45 | 0.60 | 0.12 | 0.23 | 0.80 |
| $PM_{10}$ | | | | | | | | | | | | | | | |
| Cu | | | | | | | | | | | | | | | |
| Fe | | | | | | | | 0.85 | | | | | | | |
| K | | | | | | | | 0.52 | 0.69 | | | | | | |
| Ni | | | | | | | | 0.54 | 0.68 | 0.28 | | | | | |
| S | | | | | | | | 0.41 | 0.38 | -0.001 | 0.75 | | | | |
| Si | | | | | | | | 0.44 | 0.72 | 0.84 | 0.41 | -0.07 | | | |
| V | | | | | | | | 0.44 | 0.46 | 0.07 | 0.80 | 0.77 | 0.14 | | |
| Zn | | | | | | | | 0.52 | 0.65 | 0.41 | 0.53 | 0.60 | 0.38 | 0.35 | |

Pearson Correlations had a p-value >0.001, all others p-values were<0.001.

**Table S4.** Correlations between elemental (ng/$m^3$) and total mass concentration of PM (μg/$m^3$) by cohort.

| | BAMSE | | DNBC | | ABCD | | PIAMA | | KANC | | DUISBURG | | GASPII | | INMA | |
|---|---|---|---|---|---|---|---|---|---|---|---|---|---|---|---|---|
| | $PM_{2.5}$ | $PM_{10}$ | $PM_{2.5}$ | $PM_{10}$ | $PM_{2.5}$ | $PM_{10}$ | $PM_{2.5}$ | $PM_{10}$ | $PM_{2.5}$ | $PM_{10}$ | $PM_{2.5}$ | $PM_{10}$ | $PM_{2.5}$ | $PM_{10}$ | $PM_{2.5}$ | $PM_{10}$ |
| Cu | 0.60 | 0.28 | 0.58 | 0.72 | 0.34 | 0.29 | 0.60 | 0.44 | 0.41 | 0.42 | 0.44 | 0.56 | 0.78 | 0.83 | 0.61 | 0.53 |
| Fe | 0.56 | 0.69 | 0.55 | 0.73 | 0.21 | 0.48 | 0.53 | 0.59 | na | 0.67 | 0.44 | 0.79 | 0.81 | 0.86 | 0.48 | 0.62 |
| K | 0.35 | 0.92 | 0.09 | 0.18 | 0.13 | 0.41 | 0.34 | 0.50 | na | na | na | 0.33 | 0.62 | 0.67 | 0.43 | 0.75 |
| Ni | na | 0.09 | 0.62 | 0.76 | 0.02[a] | 0.30 | 0.43 | 0.46 | na | 0.42 | 0.65 | 0.78 | 0.20 | 0.28 | 0.40 | 0.50 |
| S | 0.45 | 0.44 | 0.62 | 0.73 | 0.08 | 0.19 | 0.55 | 0.40 | na | na | 0.47 | 0.63 | 0.69 | 0.30 | 0.44 | 0.44 |
| Si | 0.42 | 0.92 | 0.66 | 0.77 | 0.14 | 0.46 | 0.51 | 0.63 | 0.05[a] | 0.11 | 0.70 | 0.65 | 0.56 | 0.54 | 0.69 | 0.76 |
| V | 0.32 | 0.68 | 0.60 | 0.71 | 0.02[a] | 0.15 | 0.41 | 0.40 | -0.02[a] | -0.03[a] | 0.39 | 0.39 | 0.34 | 0.70 | 0.35 | 0.38 |
| Zn | 0.44 | 0.60 | 0.57 | 0.73 | 0.13 | 0.38 | 0.29 | 0.30 | na | 0.26 | 0.60 | 0.59 | 0.70 | 0.85 | 0.44 | 0.22 |

Na refers to not available.

[a]Pearson Correlations had a p-value >0.001, all others p-values were<0.001.

Particle mass concentrations are pregnancy averages and particle constituents are annual averages.

**Table S5.** Sensitivity and stratified analyses of sulfur on term LBW.

| Model | N [a] | n [b] | S $PM_{2.5}$ OR | (95%CIs) | p [c] | N | n | S $PM_{10}$ OR | (95%CIs) | p [c] |
|---|---|---|---|---|---|---|---|---|---|---|
| Women who did not change address [d] | 25,765 | 300 | 1.35 | (1.14, 1.60) | | 25,765 | 300 | 1.28 | (1.12, 1.45) | |
| Study areas with highest LUR-model prediction [e] | 17,006 | 192 | 1.18 | (0.90, 1.55) | | 27,339 | 351 | 1.17 | (1.01, 1.36) | |
| Excluding DNBC | 14,949 | 223 | 1.36 | (1.11, 1.68) | | 14,949 | 233 | 1.30 | (1.10, 1.53) | |
| Women who participated once | 29,878 | 379 | 1.35 | (1.16, 1.57) | | 29,878 | 379 | 1.27 | (1.13, 1.43) | |
| Women with information on maternal ethnic origin | 29,904 | 379 | 1.32 | (1.14, 1.55) | | 29,904 | 377 | 1.25 | (1.11, 1.41) | |
| Additional adjustment for maternal ethnic origin | 29,904 | 377 | 1.30 | (1.11, 1.53) | | 29,904 | 377 | 1.23 | (1.09, 1.40) | |
| Stratified on country of birth | | | | | | | | | | |
| Women born in country of cohort | 26,714 | 309 | 1.27 | (1.07, 1.51) | | 26,714 | 309 | 1.24 | (1.02, 1.50) | |
| Women born elsewhere | 3,190 | 68 | 1.60 | (0.96, 2.64) | 0.28 | 3,190 | 68 | 1.45 | (0.99, 2.11) | 0.23 |
| Stratified on education | | | | | | | | | | |
| Women with low education | 6,029 | 119 | 1.25 | (0.92, 1.70) | | 6,029 | 119 | 1.18 | (0.93, 1.49) | |
| Women with middle education | 14,017 | 158 | 1.48 | (1.18, 1.86) | 0.67 | 14,017 | 158 | 1.50 | (1.12, 2.01) | 0.40 |
| Women with high education | 10,530 | 104 | 1.25 | (0.95, 1.65) | 0.94 | 10,530 | 104 | 1.12 | (0.90, 1.38) | 0.60 |
| Women with information on second-hand smoke exposure | 27,245 | 377 | 1.18 | (0.97, 1.45) | | 27,245 | 346 | 1.15 | (0.99, 1.33) | |
| Additional adjustment for second-hand smoke exposure | 27,245 | 346 | 1.18 | (0.96, 1.45) | | 27,245 | 346 | 1.14 | (0.98, 1.33) | |
| Stratified on maternal smoking | | | | | | | | | | |
| Non-smoking women | 26,061 | 265 | 1.27 | (1.07, 1.51) | | 26,061 | 265 | 1.21 | (1.06, 1.39) | |
| Smoking women | 4,515 | 116 | 1.47 | (1.09, 1.98) | 0.49 | 4,515 | 116 | 1.36 | (1.08, 1.71) | 0.47 |
| Stratified on sex | | | | | | | | | | |
| Women giving birth to a boy | 15,500 | 148 | 1.35 | (1.06, 1.72) | | 15,500 | 148 | 1.27 | (1.05, 1.53) | |
| Women giving birth to a girl | 15,076 | 233 | 1.36 | (1.12, 1.65) | 0.86 | 15,076 | 233 | 1.28 | (1.10, 1.48) | 0.88 |
| Stratified on parity | | | | | | | | | | |
| Primiparous women | 14,694 | 116 | 1.64 | (1.24, 2.17) | | 14,694 | 116 | 1.44 | (1.16, 179) | |
| Mulitiparous women | 15,882 | 265 | 1.26 | (1.05, 1.50) | 0.17 | 15,882 | 265 | 1.21 | (1.05, 1.39) | 0.27 |
| Stratified on age | | | | | | | | | | |
| Women aged <25 years at time of giving birth | 3,180 | 59 | 1.27 | (0.82, 1.96) | | 3,180 | 59 | 1.07 | (0.78, 1.47) | |
| Women aged 25-35 years at time of giving birth | 22,325 | 250 | 1.30 | (1.08, 1.57) | 0.13 | 22,325 | 250 | 1.23 | (1.07, 1.43) | 0.90 |
| Women aged >35 years at time of giving birth | 5,071 | 72 | 1.45 | (1.03, 2.03) | 0.81 | 5,071 | 72 | 1.44 | (1.10, 1.88) | 0.58 |
| Stratified on season of conception | | | | | | | | | | |
| Conception in January-March | 6,622 | 87 | 1.36 | (1.01, 1.84) | | 6,622 | 87 | 1.39 | (1.09, 1.77) | |
| Conception in April-June | 6,360 | 76 | 1.13 | (0.80, 1.58) | 0.59 | 6,360 | 76 | 1.03 | (0.79, 1.34) | 0.14 |
| Conception in July-September | 8,271 | 97 | 1.63 | (1.19, 2.23) | 0.53 | 8,271 | 97 | 1.41 | (1.12, 1.79) | 0.89 |
| Conception in October-December | 9,323 | 121 | 1.35 | (1.03, 1.77) | 0.93 | 9,323 | 121 | 1.28 | (1.04, 1.57) | 0.51 |

Effect estimates refer to odds ratio (OR) and 95% confidence interval (CIs) for LBW (<2,500 g) among term births (≥37 weeks of gestation) from pooled analyses using logistic regression models with random effect of centre. See table 3 for increments and adjustment. [a]N refers number of subjects in each model. [b]n refers number of cases in each model. [c]P from interaction term. [d]During pregnancy. [e]Excluding BAMSE, ABCD and PIAMA for S $PM_{2.5}$ and BAMSE for S $PM_{10}$.

**Table S6.** Sex-stratified associations between exposure to PM constituents and term LBW.

| Sex | $PM_{2.5}$ | | | | $PM_{10}$ | | | |
|---|---|---|---|---|---|---|---|---|
| Mass | N[a] | n[b] | OR[c] | (95%CIs) | N[a] | n[b] | OR[c] | (95%CIs) |
| Boys | 15,354 | 149 | 1.30 | (1.09, 1.55) | 15,354 | 149 | 1.37 | (1.12, 1.69) |
| Girls | 14,959 | 232 | 1.18 | (0.96, 1.44) | 14,959 | 236 | 1.11 | (0.88, 1.41) |
| Cu | | | | | | | | |
| Boys | 15,805 | 154 | 1.22 | (0.86, 1.73) | 15,805 | 154 | 1.26 | (0.98, 1.62) |
| Girls | 15,368 | 236 | 0.97 | (0.65, 1.43) | 15,368 | 233 | 1.02 | (0.77, 1.37) |
| Fe | | | | | | | | |
| Boys | 15,500 | 148 | 1.29 | (0.92, 1.82) | 15,805 | 154 | 1.26 | (0.96, 1.66) |
| Girls | 15,076 | 233 | 0.98 | (0.75, 1.28) | 15,076 | 236 | 0.88 | (0.61, 1.25) |
| K | | | | | | | | |
| Boys | 15,409 | 147 | 1.06 | (0.83, 1.35) | 15,500 | 148 | 0.96 | (0.76, 1.21) |
| Girls | 14,973 | 228 | 1.00 | (0.74, 1.35) | 15,973 | 233 | 0.82 | (0.62, 1.09) |
| Ni | | | | | | | | |
| Boys | 13,847 | 136 | 1.11 | (0.90, 1.36) | 15,805 | 154 | 1.17 | (0.89, 1.54) |
| Girls | 13,492 | 215 | 1.16 | (0.99, 1.36) | 15,368 | 236 | 1.50 | (1.07, 2.11) |
| S | | | | | | | | |
| Boys | 15,500 | 148 | 1.35 | (1.06, 1.72) | 15,500 | 148 | 1.27 | (1.05, 1.53) |
| Girls | 15,076 | 233 | 1.36 | (1.12, 1.65) | 15,076 | 233 | 1.28 | (1.10, 1.48) |
| Si | | | | | | | | |
| Boys | 15,805 | 154 | 0.88 | (0.62, 1.25) | 15,805 | 154 | 0.94 | (0.71, 1.24) |
| Girls | 15,368 | 236 | 0.75 | (0.49, 1.13) | 15,368 | 236 | 0.81 | (0.59, 1.10) |
| V | | | | | | | | |
| Boys | 15,805 | 154 | 1.18 | (0.92, 1.50) | 15,805 | 154 | 1.20 | (0.88, 1.65) |
| Girls | 15,368 | 233 | 1.20 | (0.89, 1.64) | 15,368 | 236 | 1.03 | (0.68, 1.55) |
| Zn | | | | | | | | |
| Boys | 15,500 | 148 | 1.21 | (0.90, 1.62) | 15,805 | 154 | 1.46 | (1.12, 1.90) |
| Girls | 15,076 | 232 | 1.26 | (0.96, 1.66) | 15,368 | 236 | 1.06 | (0.78, 1.43) |

[a]N refers to the number of subjects in each model. [b]n refers to the number of term LBW cases in each model. [c]Odds ratio (OR) and 95% confidence interval (CIs) for LBW (<2,500 g) among term births (≥37 weeks of gestation) from pooled analyses using logistic regression models with random effect of centre adjusted for gestational age, sex, parity, maternal height, pre-pregnancy weight, maternal active smoking during 2nd trimester, maternal age, maternal education and season of conception per increments of 5 μg/$m^3$ for $PM_{2.5}$; 5 ng/$m_3$ for Cu $PM_{2.5}$; 100 ng/$m^3$ for Fe $PM_{2.5}$; 50 ng/$m^3$ for K $PM_{2.5}$; 1 ng/$m^3$ for Ni $PM_{2.5}$; 200 ng/$m^3$ for S $PM_{2.5}$; 100 ng/$m^3$ for Si $PM_{2.5}$; 2 ng/$m^3$ for V $PM_{2.5}$; 10 ng/$m^3$ for Zn $PM_{2.5}$; 10 μg/$m^3$ for $PM_{10}$; 5 ng/$m_3$ for Cu $PM_{2.5}$; 10 μg/$m^3$ for $PM_{10}$; 20 ng/$m^3$ for Cu $PM_{10}$; 500 ng/$m^3$ for Fe $PM_{10}$; 100 ng/$m^3$ for K $PM_{10}$; 2 ng/$m^3$ for Ni $PM_{10}$; 200 ng/$m^3$ for S $PM_{10}$; 500 ng/$m^3$ for Si $PM_{10}$; 3 ng/$m^3$ for V $PM_{10}$; and 20 ng/$m^3$ for Zn $PM_{10}$.

**Table S7.** Associations between exposure to PM constituents and term LBW results from the study population with information on atmospheric pressure and pressure adjusted models.

| | Without adjustment for atmospheric pressure[a] | | | | | | | | With adjustment for atmospheric pressure[b] | | | | | |
|---|---|---|---|---|---|---|---|---|---|---|---|---|---|---|
| | $PM_{2.5}$ | | | | $PM_{10}$ | | | | $PM_{2.5}$ | | | $PM_{10}$ | | |
| Exposure | N | n | OR | (95%CIs) | N | n | OR | (95%CIs) | N | OR | (95%CIs) | N | OR | (95%CIs) |
| Mass | 29,716 | 372 | 1.23 | (1.05, 1.43) | 29,716 | 372 | 1.23 | (1.02, 1.48) | 29,716 | 1.25 | (1.03, 1.52) | 29,716 | 1.24 | (0.98, 1.56) |
| Cu | 30,576 | 381 | 1.08 | (0.08, 1.45) | 30,576 | 381 | 1.13 | (0.92, 1.40) | 30,576 | 1.17 | (0.89, 1.55) | 30,576 | 1.18 | (0.96, 1.46) |
| Fe | 30,576 | 381 | 1.14 | (0.92, 1.41) | 30,576 | 381 | 1.07 | (0.83, 1.37) | 30,576 | 1.17 | (0.95, 1.44) | 30,576 | 1.06 | (0.83, 1.37) |
| K | 30,382 | 375 | 1.05 | (0.82, 1.33) | 30,576 | 381 | 0.90 | (0.73, 1.11) | 30,382 | 1.21 | (0.98, 1.50) | 30,576 | 0.92 | (0.77, 1.11) |
| Ni | 27,339 | 351 | 1.14 | (1.00, 1.29) | 30,576 | 381 | 1.33 | (0.96, 1.85) | 27,339 | 0.94 | (0.74, 1.20) | 30,576 | 1.07 | (0.79, 1.46) |
| S | 30,576 | 381 | 1.36 | (1.17, 1.58) | 30,576 | 381 | 1.27 | (1.13, 1.43) | 30,576 | 1.28 | (1.05, 1.57) | 30,576 | 1.28 | (1.08, 1.52) |
| Si | 30,576 | 381 | 0.82 | (0.60, 1.12) | 30,576 | 381 | 0.90 | (0.71, 1.14) | 30,576 | 0.78 | (0.60, 1.02) | 30,576 | 0.87 | (0.72, 1.06) |
| V | 30,576 | 381 | 1.11 | (0.84, 1.46) | 30,576 | 381 | 0.98 | (0.69, 1.38) | 30,576 | 1.10 | (0.87, 1.37) | 30,576 | 1.06 | (0.79, 1.43) |
| Zn | 30,576 | 381 | 1.23 | (0.98, 1.54) | 30,576 | 381 | 1.25 | (0.98, 1.59) | 30,576 | 1.14 | (0.91, 1.41) | 30,576 | 1.18 | (0.94, 1.49) |

[a]Pooled analyses using regression models with random effect of centre adjusted for gestational age, sex, parity, maternal height, pre-pregnancy weight, maternal active smoking during 2nd trimester, maternal age, maternal education and season of conception restricted to subjects with information on atmospheric pressure (i.e. KANC is excluded). [b]Further adjusted for atmospheric pressure during full pregnancy (mBar coded as restricted cubic spline).

**Table S8.** Associations between exposure to PM constituents and term LBW results from between centers and within centers models.

| | $PM_{2.5}$ | | | | | $PM_{10}$ | | | | |
|---|---|---|---|---|---|---|---|---|---|---|
| | N[a] | n[b] | OR[c] | (95%CI) | p[d] | N[a] | n[b] | OR[c] | (95%CI) | p[d] |
| Particle mass | | | | | | | | | | |
| Overall[e] | 30,313 | 381 | 1.21 | (1.08, 1.36) | 0.94 | 30,313 | 381 | 1.22 | (1.03, 1.45) | 0.56 |
| Between centers[f] | 30,313 | 381 | 1.21 | (1.08, 1.37) | | 30,313 | 381 | 1.20 | (1.01, 1.58) | |
| Within centers[g] | 30,313 | 381 | 1.20 | (0.89, 1.63) | | 30,313 | 381 | 1.40 | (0.84, 1.54) | |
| Cu | | | | | | | | | | |
| Overall[e] | 31,173 | 390 | 1.08 | (0.81, 1.44) | 0.65 | 31,173 | 390 | 1.13 | (0.92, 1.39) | 0.81 |
| Between centers[f] | 31,173 | 390 | 1.17 | (0.76, 1.79) | | 31,173 | 390 | 1.19 | (0.75, 1.88) | |
| Within centers[g] | 31,173 | 390 | 1.02 | (0.69, 1.50) | | 31,173 | 390 | 1.12 | (0.88, 1.41) | |
| Fe | | | | | | | | | | |
| Overall[e] | 30,576 | 381 | 1.14 | (0.92, 1.41) | 0.37 | 31,173 | 390 | 1.06 | (0.83, 1.36) | 0.80 |
| Between centers[f] | 30,576 | 381 | 0.92 | (0.56, 1.52) | | 31,173 | 390 | 1.12 | (0.70, 1.81) | |
| Within centers[g] | 30,576 | 381 | 1.18 | (0.95, 1.48) | | 31,173 | 390 | 1.05 | (0.79, 1.39) | |
| K | | | | | | | | | | |
| Overall[e] | 30,382 | 375 | 1.05 | (0.82, 1.33) | 0.67 | 30,576 | 381 | 0.90 | (0.73, 1.11) | 0.92 |
| Between centers[f] | 30,382 | 375 | 1.02 | (0.78, 1.33) | | 30,576 | 381 | 0.89 | (0.68, 1.18) | |
| Within centers[g] | 30,382 | 375 | 1.16 | (0.69, 1.95) | | 30,576 | 381 | 0.91 | (0.68, 1.22) | |
| Ni | | | | | | | | | | |
| Overall[e] | 27,339 | 351 | 1.14 | (1.00, 1.29) | 0.61 | 31,173 | 390 | 1.29 | (0.96, 1.75) | 0.66 |
| Between centers[f] | 27,339 | 351 | 1.15 | (1.01, 1.32) | | 31,173 | 390 | 1.36 | (0.93, 2.00) | |
| Within centers[g] | 27,339 | 351 | 1.03 | (0.70, 1.53) | | 31,173 | 390 | 1.19 | (0.74, 1.92) | |
| S | | | | | | | | | | |
| Overall[e] | 30,576 | 381 | 1.36 | (1.17, 1.58) | 0.24 | 30,576 | 381 | 1.27 | (1.13, 1.43) | 0.72 |
| Between centers[f] | 30,576 | 381 | 1.38 | (1.19, 1.61) | | 30,576 | 381 | 1.28 | (1.14, 1.44) | |
| Within centers[g] | 30,576 | 381 | 0.87 | (0.41, 1.86) | | 30,576 | 381 | 1.18 | (0.75, 1.85) | |
| Si | | | | | | | | | | |
| Overall[e] | 31,173 | 390 | 0.83 | (0.62, 1.12) | 0.50 | 31,173 | 390 | 0.89 | (0.71, 1.13) | 0.19 |
| Between centers[f] | 31,173 | 390 | 0.77 | (0.53, 1.11) | | 31,173 | 390 | 0.77 | (0.56, 1.05) | |
| Within centers[g] | 31,173 | 390 | 0.95 | (0.58, 1.55) | | 31,173 | 390 | 1.04 | (0.76, 1.43) | |
| V | | | | | | | | | | |
| Overall[e] | 31,173 | 390 | 1.12 | (0.86, 1.44) | 0.48 | 31,173 | 390 | 1.00 | (0.72, 1.38) | 0.94 |
| Between centers[f] | 31,173 | 390 | 1.22 | (0.87, 1.70) | | 31,173 | 390 | 0.98 | (0.61, 1.58) | |
| Within centers[g] | 31,173 | 390 | 1.02 | (0.71, 1.46) | | 31,173 | 390 | 1.01 | (0.64, 1.58) | |
| Zn | | | | | | | | | | |
| Overall[e] | 30,576 | 381 | 1.23 | (0.98, 1.54) | 0.18 | 31,173 | 390 | 1.23 | (0.98, 1.53) | 0.29 |
| Between centers[f] | 30,576 | 381 | 1.43 | (1.05, 1.95) | | 31,173 | 390 | 1.45 | (1.00, 2.10) | |
| Within centers[g] | 30,576 | 381 | 1.05 | (0.76, 1.45) | | 31,173 | 390 | 1.13 | (0.86, 1.47) | |

[a]N refers to the number of subjects in each model. [b]n refers to the number of term low birth weight cases in each model. [c]Odds ratio (OR) and 95% confidence interval (CI) for low birth weight (<2,500 g) among term births (≥37 weeks of gestation) from pooled analyses using logistic regression models with random effect of centre adjusted for gestational age, sex, parity, maternal height, pre-pregnancy weight, maternal active smoking during 2[nd] trimester, maternal age, maternal education and season of conception per increments of 5 μg/m$^3$ for $PM_{2.5}$; 5 ng/m$_3$ for Cu $PM_{2.5}$; 100 ng/m$^3$ for Fe $PM_{2.5}$; 50 ng/m$^3$ for K $PM_{2.5}$; 1 ng/m$^3$ for Ni $PM_{2.5}$; 200 ng/m$^3$ for S $PM_{2.5}$; 100 ng/m$^3$ for Si $PM_{2.5}$; 2 ng/m$^3$ for V $PM_{2.5}$; 10 ng/m$^3$ for Zn $PM_{2.5}$; 10 μg/m$^3$ for $PM_{10}$; 5 ng/m$_3$ for Cu $PM_{2.5}$; 10 μg/m$^3$ for $PM_{10}$; 20 ng/m$^3$ for Cu $PM_{10}$ ; 500 ng/m$^3$ for Fe $PM_{10}$ ; 100 ng/m$^3$ for K $PM_{10}$ ; 2 ng/m$^3$ for Ni $PM_{10}$ ; 200 ng/m$^3$ for S $PM_{10}$ ; 500 ng/m$^3$ for Si $PM_{10}$; 3 ng/m$^3$ for V $PM_{10}$ ; and 20 ng/m$^3$ for Zn $PM_{10}$. [d]P-value from a test comparing the effect estimate of between center and within center. [e]Results correspond to the individual exposure estimates as reported in the main text and tables. [f]Results correspond to the centerwide mean. [g]Results correspond to the difference between individual exposure and centerwide mean exposure.

**Table S9.** Associations between exposure to PM constituents and birth weight results from the study population with information on atmospheric pressure and pressure adjusted models.

| | Without adjustment for atmospheric pressure[a] | | | | | | With adjustment for atmospheric pressure[b] | | | | | |
|---|---|---|---|---|---|---|---|---|---|---|---|---|
| | $PM_{2.5}$ | | | $PM_{10}$ | | | $PM_{2.5}$ | | | $PM_{10}$ | | |
| Exposure | N | β | (95%CIs) | N | β | (95%CIs) | N | β | (95%CIs) | N | β | (95%CIs) |
| Mass | 29,716 | -13 | (-27, 1) | 29,716 | -10 | (-24, 5) | 29,716 | -18 | (-32, -5) | 29,716 | -9 | (-24, 5) |
| Cu | 30,576 | 11 | (-7, 28) | 30,576 | 8 | (-3, 19) | 30,576 | 7 | (-10, 24) | 30,576 | 7 | (-4, 18) |
| Fe | 30,576 | 6 | (-5, 16) | 30,576 | 15 | (2, 28) | 30,576 | 5 | (-5, 15) | 30,576 | 15 | (1, 28) |
| K | 30,382 | 11 | (-11, 33) | 30,576 | 14 | (2, 27) | 30,382 | -2 | (-21, 16) | 30,576 | 13 | (1, 25) |
| Ni | 27,339 | 4 | (-15, 22) | 30,576 | 2 | (-22, 25) | 27,339 | -6 | (-18, 6) | 30,576 | 0.5 | (-22, 23) |
| S | 30,576 | -40 | (-64, -16) | 30,576 | -2 | (-21, 17) | 30,576 | -47 | (-61, -34) | 30,576 | -29 | (-41, -17) |
| Si | 30,576 | 28 | (7, 49) | 30,576 | 14 | (-1, 27) | 30,576 | 30 | (9, 50) | 30,576 | 16 | (3, 30) |
| V | 30,576 | 6 | (-13, 24) | 30,576 | 15 | (-6, 37) | 30,576 | 1 | (-17, 18) | 30,576 | 9 | (-12, 30) |
| Zn | 30,576 | -4 | (-21, 12) | 30,576 | 9 | (-4, 22) | 30,576 | -8 | (-23, 8) | 30,576 | 8 | (-5, 21) |

[a]Pooled analyses using regression models with random effect of centre adjusted for gestational age, sex, parity, maternal height, pre-pregnancy weight, maternal active smoking during 2[nd] trimester, maternal age, maternal education and season of conception restricted to subjects with information on atmospheric pressure among term births (i.e. KANC is excluded). [b]Further adjusted for atmospheric pressure during full pregnancy (mBar coded as restricted cubic spline).

**Table S10.** Associations between exposure to PM constituents and birth weight results from between centers and within centers models.

| | $PM_{2.5}$ | | | | $PM_{10}$ | | | |
|---|---|---|---|---|---|---|---|---|
| | N[a] | ß[b] | (95%CI) | p[c] | N[a] | ß[b] | (95%CI) | p[c] |
| Mass | | | | | | | | |
| Overall[d] | 30,313 | -16 | (-29, -3) | 0.02 | 30,313 | -11 | (-25, 2) | <0.01 |
| Between centers[e] | 30,313 | -48 | (-75, -20) | | 30,313 | -67 | (-105, -28) | |
| Within centers[f] | 30,313 | -10 | (-24, 5) | | 30,313 | -7 | (-21, 8) | |
| Cu | | | | | | | | |
| Overall[d] | 31,173 | 10 | (-8, 27) | 0.10 | 31,173 | 8 | (-4, 19) | 0.15 |
| Between centers[e] | 31,173 | -54 | (-132, 23) | | 31,173 | -53 | (-138, 31) | |
| Within centers[f] | 31,173 | 12 | (-5, 30) | | 31,173 | 8 | (-3, 20) | |
| Fe | | | | | | | | |
| Overall[d] | 30,576 | 6 | (-5, 16) | 0.94 | 31,173 | 14 | (1, 28) | 0.37 |
| Between centers[e] | 30,576 | 9 | (-83, 101) | | 31,173 | -28 | (-120, 65) | |
| Within centers[f] | 30,576 | 6 | (-5, 16) | | 31,173 | 15 | (2, 29) | |
| K | | | | | | | | |
| Overall[d] | 30,382 | 11 | (-11, 33) | 0.20 | 30,576 | 14 | (2, 27) | 0.92 |
| Between centers[e] | 30,382 | -19 | (-68, 31) | | 30,576 | 12 | (-42, 66) | |
| Within centers[f] | 30,382 | 18 | (-7, 42) | | 30,576 | 15 | (2, 27) | |
| Ni | | | | | | | | |
| Overall[d] | 27,339 | 4 | (-15, 22) | 0.02 | 31,173 | 1 | (-22, 24) | 0.02 |
| Between centers[e] | 27,339 | -47 | (-89, -4) | | 31,173 | -81 | (-154, -8) | |
| Within centers[f] | 27,339 | 10 | (-10, 30) | | 31,173 | 7 | (-17, 31) | |
| S | | | | | | | | |
| Overall[d] | 30,576 | -40 | (-64, -16) | 0.03 | 30,576 | -2 | (-21, 17) | <0.01 |
| Between centers[e] | 30,576 | -65 | (-94, -35) | | 30,576 | -52 | (-80, -23) | |
| Within centers[f] | 30,576 | -13 | (-48, 23) | | 30,576 | 12 | (-10, 34) | |
| Si | | | | | | | | |
| Overall[d] | 31,173 | 26 | (5, 48) | 0.78 | 31,173 | 13 | (-1, 27) | 0.18 |
| Between centers[e] | 31,173 | 37 | (-40, 114) | | 31,173 | 54 | (-7, 114) | |
| Within centers[f] | 31,173 | 26 | (4, 47) | | 31,173 | 11 | (-3, 25) | |
| V | | | | | | | | |
| Overall[d] | 31,173 | 5 | (-13, 23) | 0.04 | 31,173 | 13 | (-8, 35) | 0.60 |
| Between centers[e] | 31,173 | -65 | (-132, 1) | | 31,173 | -12 | (-107, 84) | |
| Within centers[f] | 31,173 | 9 | (-10, 27) | | 31,173 | 15 | (-7, 37) | |
| Zn | | | | | | | | |
| Overall[d] | 30,576 | -4 | (-21, 12) | 0.01 | 31,173 | 8 | (-6, 21) | <0.01 |
| Between centers[e] | 30,576 | -68 | (-118, -18) | | 31,173 | -77 | (-140, -13) | |
| Within centers[f] | 30,576 | 1 | (-17, 18) | | 31,173 | 10 | (-3, 23) | |

[a]N refers to the number of subjects in each model. [b]Coefficient and 95% confidence interval (CI) change in birth weight (g) among term births (≥37 weeks of gestation) from pooled analyses using linear regression models with random effect of centre adjusted for gestational age, sex, parity, maternal height, pre-pregnancy weight, maternal active smoking during 2[nd] trimester, maternal age, maternal education and season of conception per increments of 5 $\mu g/m^3$ for $PM_{2.5}$; 5 $ng/m_3$ for Cu $PM_{2.5}$; 100 $ng/m^3$ for Fe $PM_{2.5}$; 50 $ng/m^3$ for K $PM_{2.5}$; 1 $ng/m^3$ for Ni $PM_{2.5}$; 200 $ng/m^3$ for S $PM_{2.5}$; 100 $ng/m^3$ for Si $PM_{2.5}$; 2 $ng/m^3$ for V $PM_{2.5}$; 10 $ng/m^3$ for Zn $PM_{2.5}$; 10 $\mu g/m^3$ for $PM_{10}$; 5 $ng/m_3$ for Cu $PM_{2.5}$; 10 $\mu g/m^3$ for $PM_{10}$; 20 $ng/m^3$ for Cu $PM_{10}$ ; 500 $ng/m^3$ for Fe $PM_{10}$ ; 100 $ng/m^3$ for K $PM_{10}$ ; 2 $ng/m^3$ for Ni $PM_{10}$ ; 200 $ng/m^3$ for S $PM_{10}$ ; 500 $ng/m^3$ for Si $PM_{10}$; 3 $ng/m^3$ for V $PM_{10}$ ; and 20 $ng/m^3$ for Zn $PM_{10}$. [c]P-value from a test comparing the effect estimate of between center and within center. [d]Results correspond to the to the individual exposure estimates as reported in the main text and tables. [e]Results correspond to the centerwide mean. [f]Results correspond to the difference between individual exposure and centerwide mean exposure.

**Table S11.** Sex-stratified associations between exposure to PM constituents, birth weight and head circumference.

| | Birth weight (g) | | | | | | Birth head circumference (cm) | | | | | |
|---|---|---|---|---|---|---|---|---|---|---|---|---|
| | $PM_{2.5}$ | | | $PM_{10}$ | | | $PM_{2.5}$ | | | $PM_{10}$ | | |
| Mass | N[a] | β[b] | (95%CI) | N[a] | β[b] | (95%CI) | N[a] | β[c] | (95%CI) | N[a] | β[c] | (95%CI) |
| Boys | 15,354 | -19 | (-36, -2) | 15,354 | -16 | (-35, 2) | 10,694 | -0.20 | (-0.27, -0.13) | 10,359 | -0.20 | (-0.27, -0.13) |
| Girls | 14,959 | -22 | (-40, -5) | 14,959 | -15 | (-34, 4) | 10,694 | -0.22 | (-0.29, -0.15) | 10,359 | -0.21 | (-0.28, -0.14) |
| Cu | | | | | | | | | | | | |
| Boys | 15,805 | 26 | (2, 50) | 15,805 | 16 | (1, 32) | 11,152 | -0.27 | (-0.36, -0.18) | 10,771 | -0.30 | (-0.38, -0.21) |
| Girls | 15,368 | -12 | (-36, 12) | 15,368 | -3 | (-19, 12) | 11,152 | -0.16 | (-0.22, -0.10) | 10,771 | -0.15 | (-0.21, -0.09) |
| Fe | | | | | | | | | | | | |
| Boys | 15,500 | 13 | (-2, 28) | 15,805 | 14 | (-5, 33) | 11,152 | -0.18 | (-0.24, -0.13) | 10,771 | -0.19 | (-0.24, -0.14) |
| Girls | 15,076 | -1 | (-16, 13) | 15,076 | 14 | (-4, 33) | 11,152 | -0.19 | (-0.26, -0.11) | 10,771 | -0.16 | (-0.23, -0.09) |
| K | | | | | | | | | | | | |
| Boys | 15,409 | 3 | (-25, 31) | 15,500 | 9 | (-8, 26) | 11,152 | 0.28 | (0.17, 0.39) | 10,771 | 0.22 | (0.11, 0.33) |
| Girls | 14,973 | 12 | (-18, 42) | 15,973 | 21 | (4, 38) | 11,152 | 0.07 | (0.01, 0.13) | 10,771 | 0.01 | (-0.05, 0.07) |
| Ni | | | | | | | | | | | | |
| Boys | 13,847 | 10 | (-16, 36) | 15,805 | 6 | (-26, 38) | 9,456 | -0.48 | (-0.62, -0.34) | 9,148 | -0.63 | (-0.78, -0.48) |
| Girls | 13,492 | -13 | (-37, 12) | 15,368 | -16 | (-47, 16) | 11,152 | -0.43 | (-0.56, -0.30) | 10,771 | -0.43 | (-0.55, -0.31) |
| S | | | | | | | | | | | | |
| Boys | 15,500 | -40 | (-67, -14) | 15,500 | -1 | (-25, 24) | 11,152 | -0.59 | (-0.75, -0.43) | 10,771 | -0.79 | (-0.97, -0.62) |
| Girls | 15,076 | -62 | (-89, -35) | 15,076 | -28 | (-51, -5) | 11,152 | -0.44 | (-0.57, -0.32) | 10,771 | -0.56 | (-0.69, -0.43) |
| Si | | | | | | | | | | | | |
| Boys | 15,805 | 27 | (-1, 55) | 15,805 | 9 | (-10, 28) | 11,152 | -0.10 | (-0.20, 0.01) | 10,771 | -0.15 | (-0.26, -0.05) |
| Girls | 15,368 | 29 | (-1, 58) | 15,368 | 22 | (3, 42) | 11,152 | -0.07 | (-0.14, -0.01) | 10,771 | -0.09 | (-0.16, -0.02) |
| V | | | | | | | | | | | | |
| Boys | 15,805 | 8 | (-17, 33) | 15,805 | 20 | (-10, 50) | 11,152 | -0.47 | (-0.61, -0.32) | 10,771 | -0.38 | (-0.51, -0.24) |
| Girls | 15,368 | -7 | (-31, 18) | 15,368 | 4 | (-26, 33) | 11,152 | -0.44 | (-0.59, -0.28) | 10,771 | -0.41 | (-0.56, -0.27) |
| Zn | | | | | | | | | | | | |
| Boys | 15,500 | -4 | (-26, 19) | 15,805 | 9 | (-9, 28) | 11,152 | -0.15 | (-0.25, -0.04) | 10,771 | -0.17 | (-0.27, -0.07) |
| Girls | 15,076 | -14 | (-37, 9) | 15,368 | 2 | (-16, 20) | 11,152 | -0.24 | (-0.35, -0.14) | 10,771 | -0.28 | (-0.38, -0.18) |

[a]Coefficient and 95% confidence interval (CI) from pooled analyses using linear regression models with random effect of centre adjusted for gestational age, sex, parity, maternal height, pre-pregnancy weight, maternal active smoking during 2[nd] trimester, maternal age, maternal education and season of conception per increments of 5 $\mu g/m^3$ for $PM_{2.5}$; 5 $ng/m_3$ for Cu $PM_{2.5}$; 100 $ng/m^3$ for Fe $PM_{2.5}$; 50 $ng/m^3$ for K $PM_{2.5}$; 1 $ng/m^3$ for Ni $PM_{2.5}$; 200 $ng/m^3$ for S $PM_{2.5}$; 100 $ng/m^3$ for Si $PM_{2.5}$; 2 $ng/m^3$ for V $PM_{2.5}$; 10 $ng/m^3$ for Zn $PM_{2.5}$; 10 $\mu g/m^3$ for $PM_{10}$; 5 $ng/m_3$ for Cu $PM_{2.5}$; 10 $\mu g/m^3$ for $PM_{10}$; 20 $ng/m^3$ for Cu $PM_{10}$; 500 $ng/m^3$ for Fe $PM_{10}$; 100 $ng/m^3$ for K $PM_{10}$; 2 $ng/m^3$ for Ni $PM_{10}$; 200 $ng/m^3$ for S $PM_{10}$; 500 $ng/m^3$ for Si $PM_{10}$; 3 $ng/m^3$ for V $PM_{10}$; and 20 $ng/m^3$ for Zn $PM_{10}$. [b]Change in birth weight (g) among term births (≥37 weeks of gestation). [c]Change in birth head circumference (cm).

**Table S12.** Associations between exposure to PM constituents and birth head circumference results from the study population with information on atmospheric pressure and temperature and pressure adjusted models.

| | Without adjustment for atmospheric pressure[a] | | | | | | With adjustment for atmospheric pressure and temperature[b] | | | | | |
|---|---|---|---|---|---|---|---|---|---|---|---|---|
| | $PM_{2.5}$ | | | $PM_{10}$ | | | $PM_{2.5}$ | | | $PM_{10}$ | | |
| Exposure | N | β | (95%CIs) | N | β | (95%CIs) | N | β | (95%CIs) | N | β | (95%CIs) |
| Mass | 21,053 | -0.23 | (-0.29, -0.18) | 21,053 | -0.23 | (-0.28, -0.17) | 21,053 | -0.25 | (-0.31, -0.19) | 21,053 | -0.24 | (-0.29, -0.19) |
| Cu | 21,346 | -0.30 | (-0.36, -0.23) | 21,346 | -0.16 | (-0.20, -0.12) | 21,346 | -0.30 | (-0.36, -0.24) | 21,346 | -0.16 | (-0.21, -0.12) |
| Fe | 21,346 | -0.19 | (-0.23, -0.15) | 21,346 | -0.18 | (-0.23, -0.13) | 21,346 | -0.20 | (-0.23, -0.16) | 21,346 | -0.18 | (-0.23, -0.13) |
| K | 21,346 | 0.31 | (0.22, 0.40) | 21,346 | 0.04 | (-0.004, 0.09) | 21,346 | 0.31 | (0.22, 0.40) | 21,346 | 0.03 | (-0.01, 0.08) |
| Ni | 18,604 | -0.60 | (-0.71, -0.49) | 18,604 | -0.45 | (-0.55, -0.34) | 18,604 | -0.59 | (-0.70, -0.48) | 18,604 | -0.43 | (-0.54, -0.33) |
| S | 21,346 | -0.80 | (-0.93, -0.66) | 21,346 | -0.57 | (-0.66, -0.47) | 21,346 | -0.78 | (-0.91, -0.65) | 21,346 | -0.55 | (-0.64, -0.45) |
| Si | 21,346 | -0.17 | (-0.26, -0.09) | 21,346 | -0.11 | (-0.17, -0.06) | 21,346 | -0.17 | (-0.25, -0.09) | 21,346 | -0.11 | (-0.16, -0.06) |
| V | 21,346 | -0.45 | (-0.56, -0.35) | 21,346 | -0.48 | (-0.59, -0.38) | 21,346 | -0.46 | (-0.56, -0.35) | 21,346 | -0.49 | (-0.60, -0.38) |
| Zn | 21,346 | -0.13 | (-0.21, -0.05) | 21,346 | -0.26 | (-34, -0.19) | 21,346 | -0.13 | (-0.21, -0.05) | 21,346 | -0.25 | (-0.33, -0.18) |

[a]Pooled analyses using regression models with random effect of centre adjusted for gestational age, sex, parity, maternal height, pre-pregnancy weight, maternal active smoking during 2nd trimester, maternal age, maternal education and season of conception restricted to subjects with information on atmospheric pressure. [b]Further adjusted for atmospheric pressure during full pregnancy (mBar coded as restricted cubic spline) and temperature (°C coded as restricted cubic spline).

**Table S13.** PM constituents and birth head circumference results from between centers and within centers models.

| | $PM_{2.5}$ | | | | $PM_{10}$ | | | |
|---|---|---|---|---|---|---|---|---|
| | N[a] | ß[b] | (95%CI) | p[c] | N[a] | ß[b] | (95%CI) | p[c] |
| Mass | | | | | | | | |
| Overall[d] | 21,053 | -0.23 | (-0.29, -0.18) | <0.01 | 21,053 | -0.23 | (-0.28, -0.17) | 0.14 |
| Between centers[e] | 21,053 | -0.09 | (-0.18, 0.00) | | 21,053 | -0.14 | (-0.26, -0.03) | |
| Within centers[f] | 21,053 | -0.27 | (-0.34, -0.21) | | 21,053 | -0.25 | (-0.31, -0.18) | |
| Cu | | | | | | | | |
| Overall[d] | 21,923 | -0.29 | (-0.36, -0.23) | 0.22 | 21,923 | -0.16 | (-0.20, -0.11) | 0.81 |
| Between centers[e] | 21,923 | -0.17 | (-0.37, 0.02) | | 21,923 | -0.18 | (-0.40, 0.04) | |
| Within centers[f] | 21,923 | -0.30 | (-0.37, -0.24) | | 21,923 | -0.15 | (-0.20, -0.11) | |
| Fe | | | | | | | | |
| Overall[d] | 21,923 | -0.19 | (-0.22, -0.15) | 0.49 | 21,923 | -0.17 | (-0.22, -0.12) | 0.95 |
| Between centers[e] | 21,923 | -0.10 | (-0.35, 0.14) | | 21,923 | -0.17 | (-0.41, 0.08) | |
| Within centers[f] | 21,923 | -0.19 | (-0.23, -0.15) | | 21,923 | -0.17 | (-0.23, -0.12) | |
| K | | | | | | | | |
| Overall[d] | 21,923 | 0.30 | (0.22, 0.39) | <0.01 | 21,923 | 0.05 | (0.00, 0.09) | 0.19 |
| Between centers[e] | 21,923 | -0.06 | (-0.18, 0.06) | | 21,923 | -0.04 | (-0.18, 0.10) | |
| Within centers[f] | 21,923 | 0.35 | (0.26, 0.44) | | 21,923 | 0.05 | (0.01, 0.10) | |
| Ni | | | | | | | | |
| Overall[d] | 18,604 | -0.60 | (-0.71, -0.49) | <0.01 | 21,923 | -0.43 | (-0.53, -0.33) | 0.85 |
| Between centers[e] | 18,604 | -0.23 | (-0.43, -0.03) | | 21,923 | -0.41 | (-0.61, -0.22) | |
| Within centers[f] | 18,604 | -0.64 | (-0.75, -0.52) | | 21,923 | -0.43 | (-0.55, -0.32) | |
| S | | | | | | | | |
| Overall[d] | 21,923 | -0.79 | (-0.93, -0.66) | <0.01 | 21,923 | -0.57 | (-0.66, -0.47) | <0.01 |
| Between centers[e] | 21,923 | -0.17 | (-0.28, -0.06) | | 21,923 | -0.13 | (-0.23, -0.02) | |
| Within centers[f] | 21,923 | -0.87 | (-1.01, -0.73) | | 21,923 | -0.62 | (-0.72, -0.52) | |
| Si | | | | | | | | |
| Overall[d] | 21,923 | -0.13 | (-0.21, -0.05) | 0.59 | 21,923 | -0.09 | (-0.14, -0.04) | 0.29 |
| Between centers[e] | 21,923 | -0.08 | (-0.29, 0.14) | | 21,923 | 0.01 | (-0.17, 0.19) | |
| Within centers[f] | 21,923 | -0.14 | (-0.22, -0.06) | | 21,923 | -0.10 | (-0.15, -0.04) | |
| V | | | | | | | | |
| Overall[d] | 21,923 | -0.45 | (-0.56, -0.35) | 0.02 | 21,923 | -0.46 | (-0.57, -0.35) | <0.01 |
| Between centers[e] | 21,923 | -0.15 | (-0.40, 0.10) | | 21,923 | 0.02 | (-0.29, 0.34) | |
| Within centers[f] | 21,923 | -0.49 | (-0.60, -0.38) | | 21,923 | -0.49 | (-0.60, -0.38) | |
| Zn | | | | | | | | |
| Overall[d] | 21,923 | -0.13 | (-0.21, -0.05) | <0.01 | 21,923 | -0.25 | (-0.33, -0.18) | 0.06 |
| Between centers[e] | 21,923 | -0.31 | (-0.45, -0.17) | | 21,923 | -0.42 | (-0.60, -0.24) | |
| Within centers[f] | 21,923 | -0.09 | (-0.18, 0.00) | | 21,923 | -0.23 | (-0.31, -0.15) | |

[a]N refers to the number of subjects in each model. [b]Coefficient and 95% confidence interval (CI) for change in mean birth head circumference (cm) from pooled analyses using linear regression models with random effect of centre adjusted for gestational age, sex, parity, maternal height, pre-pregnancy weight, maternal active smoking during 2[nd] trimester, maternal age, maternal education and season of conception per increments of 5 $\mu g/m^3$ for $PM_{2.5}$; 5 $ng/m_3$ for Cu $PM_{2.5}$; 100 $ng/m^3$ for Fe $PM_{2.5}$; 50 $ng/m^3$ for K $PM_{2.5}$; 1 $ng/m^3$ for Ni $PM_{2.5}$; 200 $ng/m^3$ for S $PM_{2.5}$; 100 $ng/m^3$ for Si $PM_{2.5}$; 2 $ng/m^3$ for V $PM_{2.5}$; 10 $ng/m^3$ for Zn $PM_{2.5}$; 10 $\mu g/m^3$ for $PM_{10}$; 5 $ng/m_3$ for Cu $PM_{2.5}$; 10 $\mu g/m^3$ for $PM_{10}$; 20 $ng/m^3$ for Cu $PM_{10}$ ; 500 $ng/m^3$ for Fe $PM_{10}$ ; 100 $ng/m^3$ for K $PM_{10}$ ; 2 $ng/m^3$ for Ni $PM_{10}$ ; 200 $ng/m^3$ for S $PM_{10}$ ; 500 $ng/m^3$ for Si $PM_{10}$; 3 $ng/m^3$ for V $PM_{10}$ ; and 20 $ng/m^3$ for Zn $PM_{10}$. [c]P-value from a test comparing the effect estimate of between center and within center. [d]Results correspond to the to the individual exposure estimates as reported in the main text and tables. [e]Results correspond to the centerwide mean. [f]Results correspond to the difference between individual exposure and centerwide mean exposure.

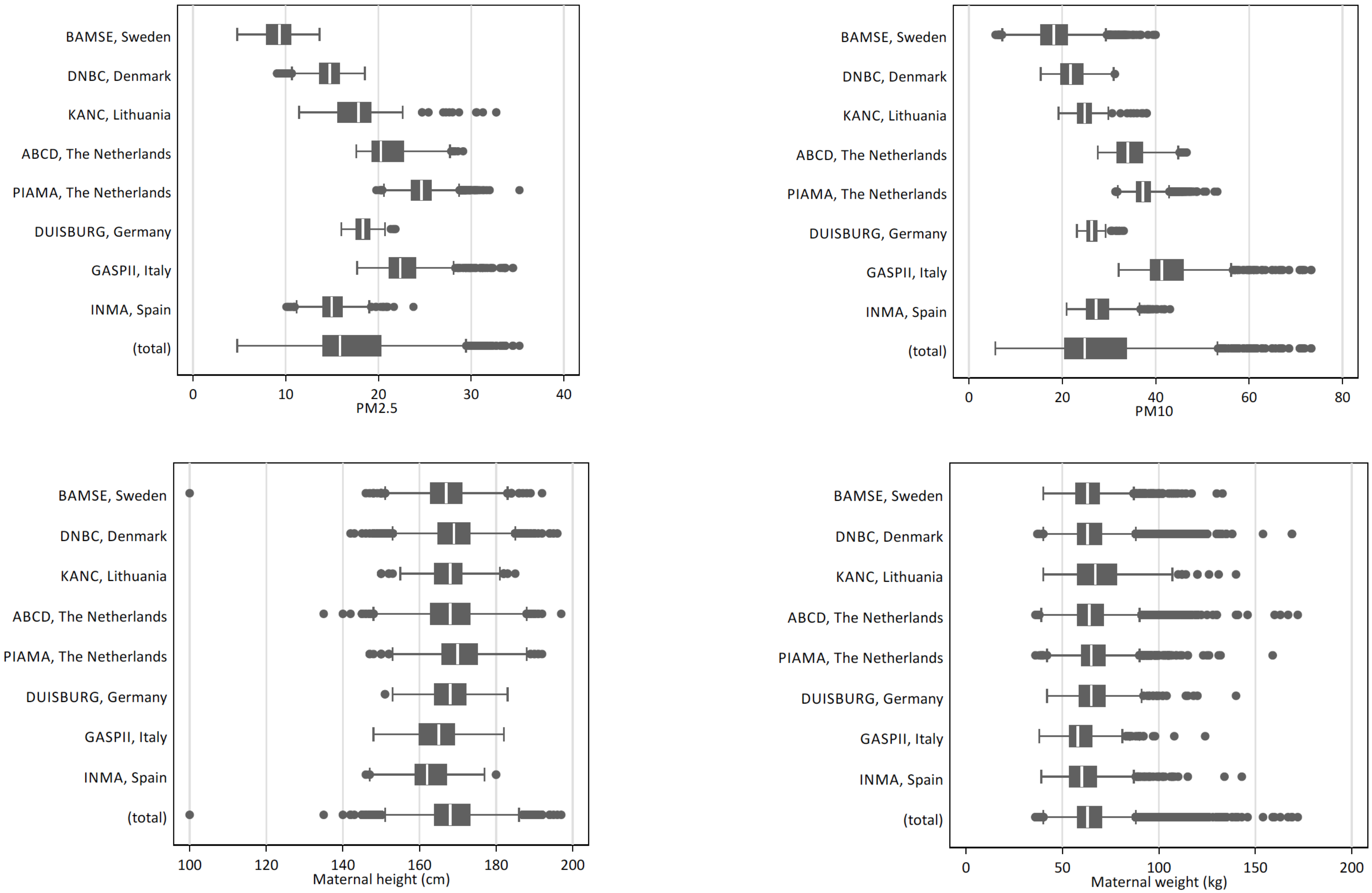
BAMSE, Sweden
DNBC, Denmark
KANC, Lithuania
ABCD, The Netherlands
PIAMA, The Netherlands
DUISBURG, Germany
GASPII, Italy
INMA, Spain
(total)
0 10 20 30 40
PM2.5
0 20 40 60 80
PM10
100 120 140 160 180 200
Maternal height (cm)
0 50 100 150 200
Maternal weight (kg)

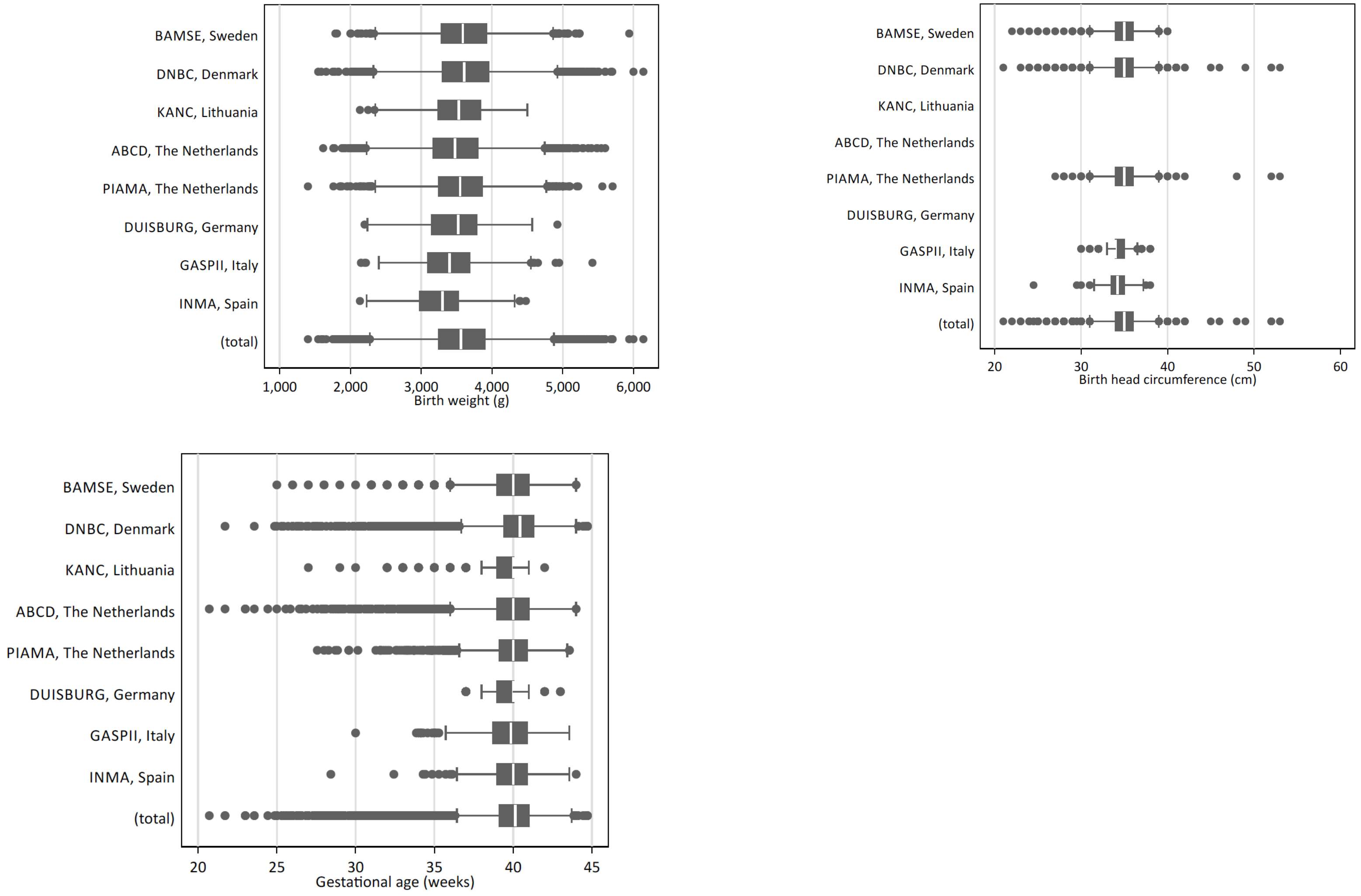


**Figure S1.** Distributions of $PM_{2.5}$, $PM_{10}$, maternal height, pre-pregnancy weight, birth weight, birth head circumference and gestational age by cohorts and for the pooled study population. The line in the middle of the box represents the median values, the ends of the box refer to the 25th and 75th percentiles and the ends of the whiskers indicate the variability outside the upper and lower quartiles (i.e., within 1.5 interquartile range of the lower quartile and upper quartile). Outliers are plotted as individual dots.
